# Body mass index and tuberculosis risk: an updated systematic literature review and dose–response meta-analysis

**DOI:** 10.1093/ije/dyaf154

**Published:** 2025-09-08

**Authors:** Matthew J Saunders, J Peter Cegielski, Rebecca A Clark, Rein M G J Houben, C Finn McQuaid

**Affiliations:** Institute for Infection and Immunity, City St George’s, University of London, London, United Kingdom; Faculty of Public Health and Policy, London School of Hygiene & Tropical Medicine, London, United Kingdom; Department of Epidemiology, Rollins School of Public Health, Emory University, Atlanta, United States; Department of Infectious Disease Epidemiology and Dynamics, London School of Hygiene & Tropical Medicine, London, United Kingdom; Department of Infectious Disease Epidemiology and Dynamics, London School of Hygiene & Tropical Medicine, London, United Kingdom; Department of Infectious Disease Epidemiology and Dynamics, London School of Hygiene & Tropical Medicine, London, United Kingdom

**Keywords:** tuberculosis, systematic review, body mass index, undernutrition, undernourishment, dose–response meta-analysis

## Abstract

**Background:**

The relationship between nutritional status and tuberculosis is critically important but poorly understood. We extended a 2009 review characterizing the relationship between body mass index (BMI) and tuberculosis risk.

**Methods:**

We systematically searched for new studies published between 2009 and 2024 investigating BMI and tuberculosis risk in adults. We extracted estimates of risk in BMI categories, used resampling to assign a median BMI ‘dose’ within each category, and included these in one-stage dose–response meta-analyses, stratifying results by population group and country tuberculosis burden. We fitted linear models for comparability with the 2009 review and restricted cubic spline models to investigate nonlinear relationships and piecewise linear models.

**Results:**

Our analyses showed an inverse dose–response relationship between BMI and tuberculosis risk across all populations in the full underweight to obese range (15.0–35.0 kg/m^2^). The spline and piecewise linear models showed a nonlinear relationship—in 22 general-population cohorts (*n* = 24 921 531), there was a steep per-unit reduction in risk for BMI of <25.0 kg/m^2^ [18.0%, 95% confidence interval (CI): 16.4–19.6], which decreased more gradually for BMI of ≥25.0 kg/m^2^ (6.9%, 95% CI: 4.6–9.2). In 18 cohorts of people with HIV (*n* = 162 609), the reduction was 15.3% for BMI of <23.0 kg/m^2^ (95% CI: 13.1–17.5) and 2.6% (95% CI: –3.1–7.9) for BMI of ≥23.0 kg/m^2^. In three cohorts of people with diabetes (*n* = 1 118 424), the reduction was 20.5% for BMI of <24.0 kg/m^2^ (95% CI: 18.4–22.6) and 13.4% (95% CI: 3.9–22.0) for BMI of ≥24.0 kg/m^2^. Based on the global BMI distribution, we estimated a relative risk of tuberculosis associated with undernutrition (BMI < 18.5 kg/m^2^) of 5.0 (95% CI: 4.2–5.9).

**Conclusion:**

Our results highlight the independent importance of nutritional status as a driver of the tuberculosis epidemic.

Key Messages

- We aimed to address limitations of previous reviews and comprehensively characterize the relationship between body mass index (BMI) and tuberculosis risk in adults.
- We demonstrated an inverse dose–response nonlinear relationship between BMI and tuberculosis risk in the full underweight to obese range (15.0–35.0 kg/m^2^), in general-population cohorts, people with HIV, and people with diabetes across a range of countries with high and lower tuberculosis burden.
- Taken together, the empirical evidence demonstrates the fundamental independent importance of nutritional status as a determinant of tuberculosis, highlighting the need for social protection and other interventions to improve nutrition as part of a holistic global tuberculosis response.

## Introduction

The global tuberculosis epidemic remains a leading cause of poor health and wellbeing [1]. In 2023, tuberculosis affected an estimated 10.8 million people and killed 1.25 million, >80% of whom lived in low- and middle-income countries (LMIC) [2]. Although the incidence has declined since 2010, the pace of the decline is inadequate to achieve World Health Organization (WHO) End TB Strategy targets and has been partially reversed by COVID-19 [2]. One potential reason for this is the lack of action on addressing social determinants such as undernutrition, which have been demonstrated consistently to be key drivers of tuberculosis incidence [3].

Both the WHO End TB Strategy and the United Nations Sustainable Development Goals conceptualize tuberculosis as a development challenge and call for poverty reduction interventions and action on social determinants, in addition to the equitable expansion of biomedical interventions [4]. Undernutrition is the leading driver of tuberculosis, responsible for ∼10% of cases in 2023, which is higher than HIV, alcohol use disorders, smoking, and diabetes [2, 5]. In countries with a high prevalence of undernutrition, including many with a high HIV prevalence, the population attributable fraction (PAF) of tuberculosis due to undernutrition is considerably higher [6].

Although undernutrition is generally considered to be a binary variable for calculating a PAF, this is a simplification of a complex relationship between nutritional status and tuberculosis. A 2009 review of six prospective studies demonstrated an inverse log-linear relationship between body mass index (BMI, expressed as kg/m^2^)—a widely used indicator of nutritional status—and tuberculosis risk in the normal to overweight BMI range (18.5–30.0 kg/m^2^): the tuberculosis risk declined by 13.8% per unit increase in BMI [7]. This was limited to high-income countries (HIC) with a lower tuberculosis burden and only crudely illustrated uncertainty in tuberculosis risk, but not BMI. A more recent Cochrane review investigating the risk of tuberculosis associated with undernutrition (defined by an underweight BMI < 18.5 kg/m^2^) included studies from a wider range of settings but did not further investigate the log-linear relationship demonstrated previously and has methodological limitations that raise concerns around bias [8].

In this study, we aimed to address the limitations of previous reviews and comprehensively characterize the relationship between nutritional status (defined through BMI) and tuberculosis risk in adults.

## Methods

### Search strategy and selection criteria

We systematically reviewed the literature for studies investigating the relationship between BMI and tuberculosis risk. As the 2009 review [7] covered literature published until December 2008, we searched Medline and Embase for additional studies published between 1 January 2009 and 28 March 2024. Search terms were developed in consultation with a librarian (Supplementary Table S1). We also screened the references of included articles, two related reviews [9, 10], and the Cochrane review [8], and sought expert opinion on potential studies from our collaborators, including after peer review.

We included prospective and retrospective cohort studies that reported height and weight or BMI at baseline and had tuberculosis as the outcome. We applied no restrictions on publication status or language. We excluded case–control studies (unless nested within a cohort); studies with self-reported tuberculosis, tuberculosis mortality, or *Mycobacterium tuberculosis* infection as the outcome; studies only undertaken in people aged <18 years because of differences in anthropometric measures and tuberculosis diagnosis; studies performed in specific populations with limited generalizability (e.g. post gastrectomy); conference abstracts later published as full articles; and studies with insufficient data to include in meta-analyses.

Two authors (M.J.S. and J.P.C.) reviewed the full texts of studies included in the 2009 review [7], as well as those returned by the updated search after removing duplicates and screening titles and abstracts. Further details on the selection process are described in the Supplementary Methods.

### Data analysis

Data were extracted from published manuscripts and supplementary materials into an extraction form by M.J.S. and J.P.C. We classified studies by population (general population, people with HIV, and people with diabetes) and whether they were conducted in settings with a high tuberculosis burden or a lower tuberculosis burden, as defined by WHO [2]; we stratified our analyses by population and country tuberculosis burden (only possible for the general-population studies). For all studies, we extracted data on the number and type of study participants, the years of recruitment, the mean duration of follow-up per participant, how tuberculosis was defined and ascertained, and which variables were controlled for in adjusted analyses. We did not quantitatively assess the risk of bias or clinical/methodological heterogeneity in the included studies. Analyses were performed by using RStudio (version 2023.03.1 + 446) and all *P* values generated were two-sided.

To investigate the relationship between BMI and tuberculosis risk, we conducted one-stage dose–response meta-analyses [11]. To do this, we extracted estimates of the tuberculosis incidence rate ratios (IRRs) or hazard ratios (HRs) for the BMI categories reported in each study compared with a reference category (e.g. <18.5 versus 18.5–25.0 kg/m^2^), with their 95% confidence intervals (CIs) and the associated number of events and person-years. For pooling estimates, we considered the IRRs and HRs to be approximately equivalent and, from here on, refer to them as relative risks (RRs) [12]. To minimize the effect of confounders, we used adjusted estimates wherever possible. To select a midpoint BMI ‘dose’ corresponding to each RR for each BMI category, we used the median of 1000 randomly drawn BMI values for each BMI category in each study, assuming that the BMI in the overall study population followed a log-normal distribution (Supplementary Methods and Supplementary Box S1).

We calculated the natural logarithms of the extracted RRs and 95% CIs, and estimated the standard error (SE) by using the formula: SE=[log(upper 95% CI) – log(lower 95% CI)]/3.92. We then fitted random-effects models by using the ‘dosresmeta’ package in R with a restricted maximum-likelihood estimator and using the Greenland and Longnecker method for approximating the covariance matrix based on the number of events and person-years in each BMI category [11]. For direct comparability with the 2009 review, we fitted a prespecified linear model for each population group to estimate the percentage reduction in tuberculosis risk per unit increase in BMI [7]. Then, because we hypothesized that the relationship would be nonlinear, with the tuberculosis risk increasing the most at lower BMI values, we fitted a restricted cubic spline model with knots set at the 10th, 50th, and 90th percentiles of the BMI ‘dose’ and compared the model fit by using the Akaike Information Criterion (AIC) and Bayesian Information Criterion (BIC). Finally, to provide a readily interpretable and usable output—the percentage reduction in tuberculosis risk per unit increase in BMI in different BMI ranges—we fitted a piecewise linear model with a single breakpoint for each population group, based on the shape of the spline model. We identified population-specific optimal breakpoints by testing the prespecified breakpoint BMI values between 18.5 and 25.0 kg/m^2^ (because we hypothesized that the change in risk would occur in the normal weight range) and used the whole-number value that minimized the AIC/BIC values.

We created plots visualizing these models and extracted estimates for the RRs of tuberculosis associated with undernutrition (defined by an underweight BMI < 18.5 kg/m^2^). For comparability with previous RR estimates, we performed this for all population groups by comparing BMIs of 16.0 versus 25.0 kg/m^2^ [6]. For general-population cohorts, we also extracted estimates for the RR based on the global BMI distribution by comparing the estimated mean BMI between underweight (<18.5 kg/m^2^) people versus non-underweight (≥18.5 kg/m^2^) people, and versus the midpoint of the normal weight range (21.8 kg/m^2^) (Supplementary Methods).

## Results

**Figure 1.**
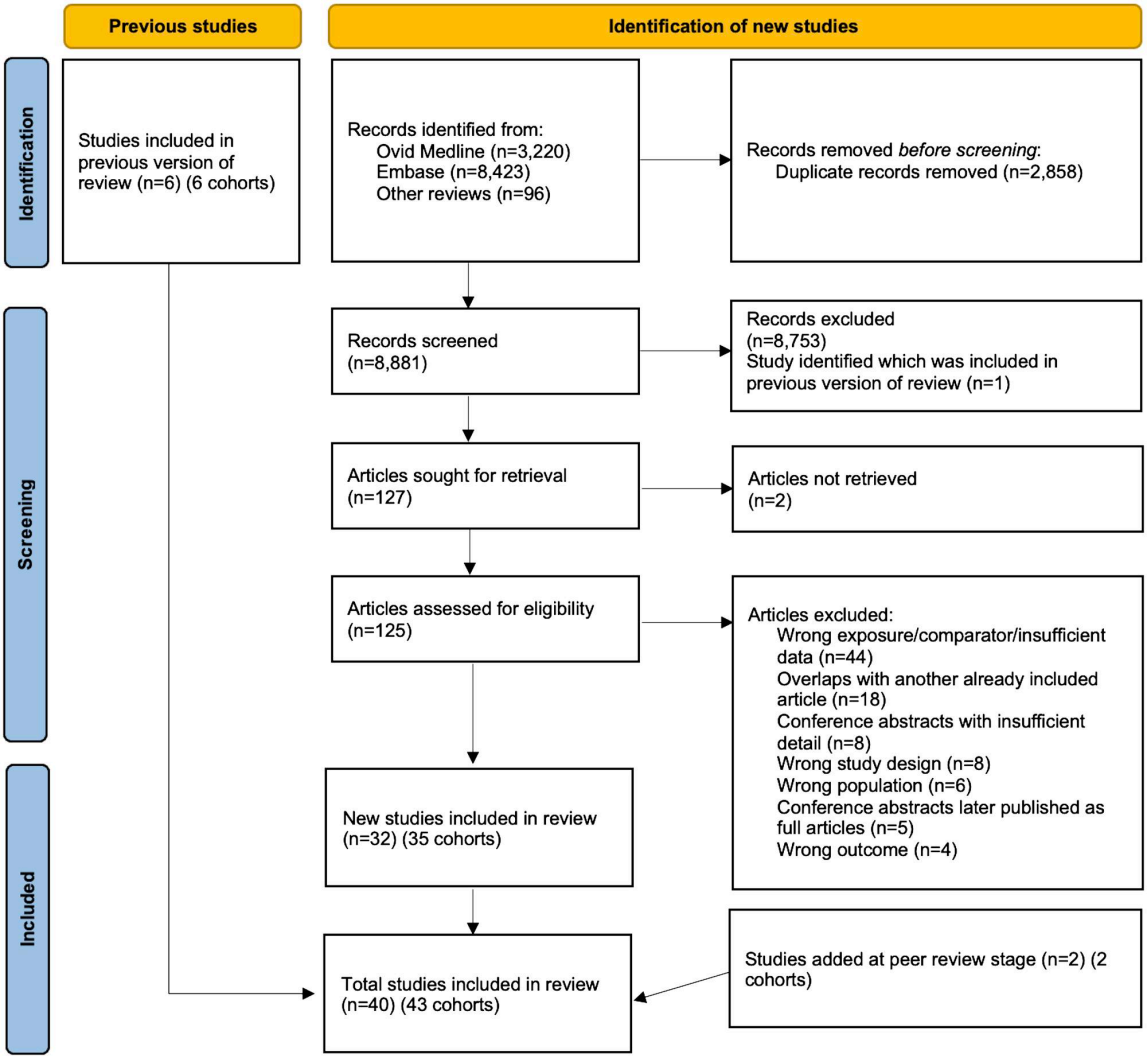
Study selection.

**Figure 2.**
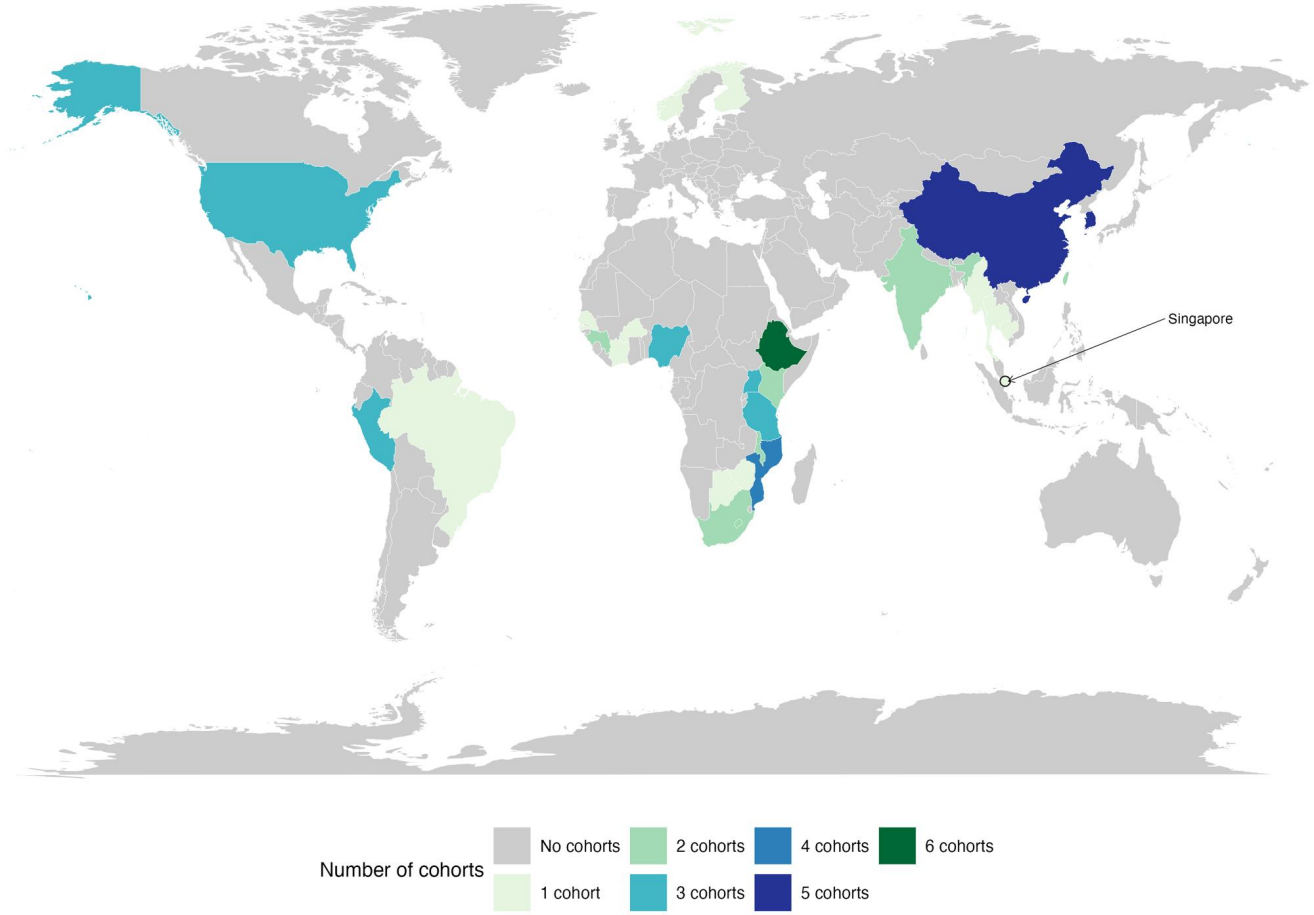
Geographic distribution of included cohorts. Some cohorts included participants from multiple countries; see Table 1.

After the removal of duplicates, our search yielded 8881 records, of which 127 articles were sought for full-text review. Forty studies describing 43 cohorts were included—34 (37 cohorts) identified in our new search and 6 from the 2009 review (Figure 1). All were cohort studies (some embedded in randomized trials), with recruitment undertaken between 1949 and 2021. An overview of cohort characteristics is shown in Table 1 and the geographic distribution is shown in Figure 2.

**Table 1.** Characteristics of included cohorts classified by population group and ordered by WHO region and country.

Study	TB burden status	Country	WHO region	Years of recruitment	Main exclusions	Population	Number of participants^a^	Average follow-up time (years)	Outcome	Ascertainment	Variables adjusted for
**General-population cohorts**											
Larsson *et al.* 2025 [40]	High burden	Zimbabwe, Tanzania, Mozambique	AFR	2021	TB diagnosed during first 30 days	Household contacts of people with microbiologically confirmed TB	2107	2 (median)	All forms of TB	Active follow-up every 6 months	Site, age, sex, HIV
Saunders *et al.* 2017 [33]	High burden	Peru	AMR	2002–6	TB diagnosed during the first 30 days after index case enrolment	Household contacts aged ≥18 years of people with pulmonary TB	1767	2.8	All forms of TB	Active case finding, prevalence surveys, and clinic registers	Age, sex, poverty, previous TB, diabetes
Saunders *et al.* 2020 [32]	High burden	Peru	AMR	2007–15	TB diagnosed during the first 30 days after index case enrolment	Household contacts aged ≥18 years of people with all forms of TB	11 605	2.9	All forms of TB	Active case finding, prevalence surveys, and clinic registers	Age, sex, poverty, previous TB
Aibana *et al.* 2016 [27]	High burden	Peru	AMR	2009–12	TB diagnosed during the first 15 days after index case enrolment	Household contacts aged ≥18 years of people with pulmonary TB	7606	1.0	All forms of TB	Active follow-up 2, 6, and 12 months after recruitment	Age, sex, poverty, diabetes, HIV, smoking, alcohol use, IPT
Palmer *et al.* 1957 [31]	Lower burden	USA	AMR	1949–51	Current TB	Male navy recruits aged 17–21 years	68 754	4.0	All forms of TB	Annual chest radiography, contact screening, tuberculin surveys, and hospital records	Age and sex, and tuberculin reactivity implicitly adjusted for
Edwards *et al.* 1971 [30]	Lower burden	USA	AMR	1958–67	None	Male navy recruits aged 17–21 years	823 199	4.0	All forms of TB	Annual chest radiography, contact screening, tuberculin surveys, and hospital records	Age and sex, and tuberculin reactivity implicitly adjusted for
Cegielski *et al.* 2012 [29]	Lower burden	USA	AMR	1971–5	Previous TB	People aged 25–74 years participating in the National Health and Nutrition Examination Survey (NHANES 1) and associated follow-up study (NHEFS)	14 189	15.8	All forms of TB	Interviews, medical records, death certificates	Age, sex, race, income, smoking, skinfold thickness, muscle area, diabetes, Hb, hypoalbuminaemia, iron status
Hemila *et al.* 1999 [25]	Lower burden	Finland	EUR	1985–93	History of severe comorbidities; TB diagnosed in hospitals before randomization; TB diagnosed in general practice without hospital confirmation	Males aged 50–69 years who smoked and were participating in a trial on the effect of vitamin C supplementation on cancer	26 975	6.4	All forms of TB	National hospital discharge register	Age, sex (implicitly), marital status, education, residential area, smoking, alcohol use, vitamin supplements
Tverdal 1986 [26]	Lower burden	Norway	EUR	1963–75	None	People aged ≥15 years participating in the national mass radiography service	1 717 655	12.0	All forms of TB, but only new cases	National TB surveillance system	Age, sex
Paradakar *et al.* 2020 [51]	High burden	India	SEA	2014–17	TB diagnosed at enrolment	Household contacts aged ≥18 years of people with pulmonary TB	647	1.6	All forms of TB	Active follow-up after 6, 12, and 24 months	Age, sex, HIV, smoking, alcohol consumption, LTBI, index case age, index sex, index HIV, index smear/culture, household type, income, where index slept
Sinha *et al.* 2024 [49]	High burden	India	SEA	2015–19	TB diagnosed at enrolment	Household contacts of any age of people with pulmonary TB	857	2.0	All forms of TB	Unknown	Age, sex, HIV
Chen *et al.* 2022 [13]	High burden	China	WPR	2013	TB diagnosed at enrolment	People aged ≥15 years participating in a community TB screening cohort	26 022	1.7	All forms of TB	Yearly active follow-up	Age, sex, ethnicity, marital status, previous TB, smoking, alcohol use, known diabetes
Cheng *et al.* 2020 [14]	High burden	China	WPR	2013	TB diagnosed at enrolment	People aged ≥65 years who participated in a baseline TB prevalence survey	34 076	1.3	Bacteriologically confirmed TB	Yearly active follow-up and National TB surveillance system	Age, sex, smoking
Jiang *et al.* 2024 [18]	High burden	China	WPR	2016	TB diagnosed at enrolment	People aged ≥65 years participating in a basic health survey	39 122	6.8	All forms of TB	National TB surveillance system	Age, sex, smoking, alcohol use, physical activity
Leung *et al.* 2007 [20]	Lower burden	Hong Kong	WPR	2000	TB diagnosed during the first 3 months of follow-up	People aged ≥65 years enrolled at 18 health centres for elderly populations	42 116	5.0	All forms of TB	Death registry and TB notification registry	Age, sex, smoking, alcohol use, language, marital status, education, housing, work status, receiving public assistance, diabetes, cholesterol, cardiovascular disease, hypertension, COPD, asthma, malignancy, recent weight loss, recent hospital admission, ADL scores
Soh *et al.* 2019 [23]	Lower burden	Singapore	WPR	1993–8	Previous TB	People aged between 45 and 74 years belonging to two of the major dialect groups of Chinese in Singapore	50 398	16.9	All forms of TB	National TB surveillance system	Age, sex, year of recruitment, language, education, smoking, alcohol use, tea intake, energy intake, protein intake, cholesterol and fatty acids, vitamin A/C, diabetes
Choi *et al.* 2021 [16] (no diabetes)	Lower burden	South Korea	WPR	2009	TB diagnosed before enrolment	People aged ≥20 years who participated in a national health screening exam in 2009	9 204 330	7.3	All forms of TB	Korean National Health Insurance system/rare intractable disease programme	Age, sex, smoking, alcohol use, physical activity, income, hypertension, dyslipidaemia, diabetes (implicitly)
Cho *et al.* 2022 [15]	Lower burden	South Korea	WPR	2010	TB diagnosed before 2010; TB diagnosed during first year of follow-up	People aged ≥20 years who participated in a national health screening exam in 2010	11 135 332	6.3	All forms of TB	Korean National Health Insurance system/rare intractable disease programme	Age, sex, smoking, alcohol use, physical activity, income, hypertension, diabetes, dyslipidaemia
Kim *et al.* 2018 [19]	Lower burden	South Korea	WPR	2002–6	People with HIV; TB diagnosed before 2007	People aged 20–89 years in 2007 who participated in national health screening exams between 2002 and 2006	301 081	6.4	All forms of TB	Korean National Health Insurance system/rare intractable disease programme	Age, sex, household income, smoking, alcohol use, diabetes
Yoo *et al.* 2021 [24]	Lower burden	South Korea	WPR	2009–14	Previous TB	People aged 66 years who participated in national health screening exams between 2009 and 2014	1 245 640	6.5	All forms of TB	Korean National Health Insurance system/rare intractable disease programme	Age (implicitly), sex, income, smoking, alcohol use, physical activity, Hb, GFR, TUG test, diabetes, ischaemic heart disease, stroke, pulmonary disease
Lin *et al.* 2018 NHIS [22]	Lower burden	Taiwan	WPR	2001–9	Previous TB	Adults with median age 42 years who participated in cross-sectional national health surveys	48 713	7.6	Bacteriologically confirmed TB	National TB surveillance system	Age, sex, marital status, education, smoking, alcohol use, employment status, household income
Lin *et al.* 2018 NTC [22]	Lower burden	Taiwan	WPR	2005–8	Previous TB	Adults with median age 51 years who participated in a community-based voluntary health screening programme	119 340	7.3	Bacteriologically confirmed TB	National TB surveillance system	Age, sex, marital status, education, smoking, alcohol use
**People with HIV**											
Mupfumi *et al.* 2018 [44]	High burden	Botswana	AFR	2008–11	TB diagnosed at enrolment	People aged ≥18 years with an AIDS defining condition or a CD4 count <250 eligible for ART	240	1.7	All forms of TB	Clinic visits at 1 month then every 3 months until Week 96	Hb
Tchakounte Youngui *et al.* 2020 [47]	Mixed high and lower burden	Burkina Faso, Senegal, and Ivory Coast	AFR	2010–16	TB diagnosed 6 months before enrolment until 7 days after enrolment	People with HIV aged ≥16 years who started ART and had at least one follow-up visit	4249	0.83	All forms of TB	Clinic registers	Centre, age, sex, CD4 count, Hb
Alemu *et al.* 2020 [36]	High burden	Ethiopia	AFR	2013	People with TB at enrolment	People with HIV aged ≥15 years newly registered at ART centres of seven public health facilities	566	3.8	All forms of TB	Clinic registers	Sex, employment, marital status, family size, alcohol use, previous TB, functional status, WHO stage, CD4 count, Hb, ART, IPT, cotrimoxazole
Tiruneh *et al.* 2019 [48]	High burden	Ethiopia	AFR	2009–12	TB diagnosed at enrolment	People with HIV aged ≥18 years enrolled on ART at two health facilities in Western Ethiopia	600	2.5	Not specified	Clinic registers	Age, CD4 count, weight, WHO stage, functional status, cotrimoxazole, opportunistic infection, IPT
Ahmed *et al.* 2018 [35]	High burden	Ethiopia	AFR	2010–11	People on TB treatment at enrolment	People with HIV aged ≥15 years newly enrolled in HIV care in government health facilities	451	3.1	All forms of TB	Clinic registers	Marital status, family size, substance use, previous TB, opportunistic infection, bedridden, length of follow-up, WHO stage, Hb, CD4 count, IPT
Aemro *et al.* 2020 [34]	High burden	Ethiopia	AFR	2014–18	TB diagnosed at enrolment	People with HIV aged ≥15 years commencing ART in a referral hospital	494	2.0	All forms of TB	Clinic registers	Previous TB, HIV disclosure, WHO stage, CD4 count, Hb, functional status, ART adherence, IPT
Nicholas *et al.* 2011 [46] (during ART)	Mixed high and lower burden	Guinea, Kenya, Malawi, Mozambique, Nigeria, Uganda	AFR	2006–8	TB diagnosed at enrolment or during first 15 days after ART initiation	People with HIV with median age 34 years enrolled in MSF-supported HIV programmes, during ART	19 325	0.90	All forms of TB	Clinic registers	Setting, year, sex, age, prior ART, previous TB, CD4 count, time since ART
Nicholas *et al.* 2011 [46] (pre ART)	Mixed high and lower burden	Guinea, Kenya, Malawi, Mozambique, Nigeria, Uganda	AFR	2006–8	TB diagnosed at enrolment or during first 15 days after enrolment	People with HIV with median age 34 years enrolled in MSF-supported HIV programmes, pre-ART	8998	0.82	All forms of TB	Clinic registers	Setting, sex, previous TB, CD4 count
Chang *et al.* 2015 [37]	High burden	Nigeria	AFR	2005–10	TB diagnosed 6 months before enrolment until 3 months after ART initiation	People with HIV aged ≥15 years commencing ART at the Harvard/APIN PEPFAR programme	18 142	2.5	All forms of TB	Clinic registers	Sex, ART enrolment year, ART regimen, WHO stage, CD4 count, viral load, Hb, ART adherence
Nguenha *et al.* 2025 [45]	High burden	South Africa, Mozambique, Ethiopia	AFR	2016–17	People who had TB at baseline	People aged ≥18 years participating in a randomized–controlled trial	3593	1.7	All forms of TB	Active follow-up at 12 and 24 months	Age and site
Kufa *et al.* 2016 [39]	High burden	South Africa	AFR	2011–12	TB diagnosed before enrolment or during the first 3 months of follow-up	People with HIV aged 18–45 years with a recent CD4 count >350 participating in a randomized trial of a TB vaccine	634	0.9	All forms of TB	Active follow-up at 6 and 12 months	ART
Maro *et al.* 2010 [42]	High burden	Tanzania	AFR	2001–5	TB diagnosed at enrolment	People with HIV aged ≥18 years identified through HIV testing centres who were placebo recipients in a randomized trial of a TB vaccine	979	3.2	All forms of TB	Clinic visits at 2, 4, and 6 months, and then every 6 months	No adjusted analyses available
Maokola *et al.* 2021 [41]	High burden	Tanzania	AFR	2012–16	People who took IPT; people with LTBI; TB diagnosed before or at enrolment	People aged ≥20 years enrolled in 315 care and treatment clinics for HIV	75 812	1.7	All forms of TB	Clinic registers	Age, sex, ART, functional status, WHO stage
Moore *et al.* 2007 [43]	High burden	Uganda	AFR	2003–5	TB diagnosed at enrolment	People aged ≥18 years eligible for ART participating in a randomized trial	1029	1.3	All forms of TB	Active case finding—weekly home visits	Age, sex, CD4 count, viral load, participated in a previous study, previous TB
Batista *et al.* 2013 [28]	High burden	Brazil	AMR	2007–10	TB diagnosed before enrolment or during the first month of follow-up	People with HIV aged ≥18 years receiving treatment in two referral hospitals	2020	2.5	All forms of TB	Clinic registers, record linkage with TB surveillance system, deaths from TB from mortality surveillance system	Age
Kyaw *et al.* 2022 [52]	High burden	Myanmar	SEA	2011–17	TB diagnosed before enrolment; people who took IPT	People aged ≥15 years enrolled in the integrated HIV care programme	20 865	2.6	All forms of TB	Clinic registers	No adjusted analyses available
Salvadori *et al.* 2015 [50]	High burden	Thailand	SEA	1999–2012	None noted	People with HIV (adults, age unspecified) enrolled in the programme for HIV prevention and treatment	1682	7.0	All forms of TB	Clinic visits every 6 months	Age, sex, year, viral load, Hb, CD4 count
Choun *et al.* 2013 [17]	High burden	Cambodia	WPR	2003–10	People on TB treatment at enrolment, included but after they finished treatment for that episode	People aged ≥18 years enrolled in the HIV care programme in a referral hospital	2930	2.7	All forms of TB occurring within the first 6 months of ART	Clinic registers	Previous TB, On TB treatment at enrolment, sex, age, Hb, CD4 count
**People with diabetes**											
Gedfew *et al.* 2020 [38]	High burden	Ethiopia	AFR	2013–17	TB diagnosed before diagnosis of diabetes	People with diabetes aged ≥18 years registered for chronic follow-up care in a referral hospital	433	2.5	Not specified	Clinic registers	Type of diabetes, previous TB, diabetes medications, alcohol use
Li *et al.* 2020 [21]	High burden	China	WPR	2010–15	TB diagnosed before diagnosis of diabetes	People with type 2 diabetes aged ≥35 years identified through the Shanghai Standardized Diabetes Management System	234 418	3.6	Pulmonary TB (positive smear, culture, or pulmonary lesions confirmed by pathological examination) only	Shanghai TB surveillance system	No adjusted analyses available
Choi *et al.* 2021 [16] (diabetes)	Lower burden	South Korea	WPR	2009	TB diagnosed before enrolment	People aged ≥20 years who participated in a national health screening exam in 2009	883 573	7.1	All forms of TB	Korean National Health Insurance system/rare intractable disease programme	Age, sex, smoking, alcohol use, physical activity, income, hypertension, dyslipidaemia

AFR, African Region; AMR, Region of the Americas; ART, antiretroviral therapy; CRP, C reactive protein; EUR, European Region; Hb, haemoglobin; IPT, isoniazid preventive treatment; GFR, glomerular filtration rate; LTBI, latent TB infection; MSF, Médecins sans Frontières; NHIS, National Health Interview Surveys; NTC, New Taipei City; SEA, South-East Asian Region; TUG, timed up-and-go; TB, tuberculosis; WPR, Western Pacific Region.

a Where reported, this is the total number of people included in the analysis (as some participants are typically excluded from multivariable models due to missing data).

The total sample size was 26 202 564. There were 14 cohorts in the WHO Western Pacific Region (*n* = 23 367 091; 89% of the total population), dominated by 2 large studies in South Korea [13–24], 2 in the European Region (*n* = 1 744 630; 6.7%) [25, 26], 7 in the Region of the Americas (*n* = 929 140; 3.5%) [27–33], 16 in the African Region (*n* = 137 652; 0.53%) [34–48], and 4 in the South-East Asian region (*n* = 24 051; 0.09%) [49–52]. No eligible cohorts were identified from the Eastern Mediterranean Region. There were important differences in populations, which ranged from general-population-based cohorts among healthy individuals (mostly in the Western Pacific Region) [13–16, 18–20, 22–26, 29–31] to specific cohorts of people with HIV (mostly in the African Region) [17, 28, 34–37, 39, 41–48, 50, 52]. There were also six cohorts of household contacts of people with tuberculosis (three in Peru, two in India, and one in Southern Africa) [27, 32, 33, 40, 49, 51], which we grouped with general-population cohorts, and three cohorts of people with diabetes [16, 21, 38]. Most cohorts used a broad definition of all forms of tuberculosis as the outcome, which included both pulmonary and extra-pulmonary tuberculosis diagnosed either microbiologically or clinically/radiologically. Tuberculosis was mostly diagnosed by following local protocols and practices, and ascertained by researchers using clinic registers and by linking identification data to national surveillance systems. Some studies undertook active follow-up that included home visits and tuberculosis testing. Tuberculosis diagnosed at baseline or early during follow-up was mostly excluded from the analyses. The mean follow-up time per participant ranged from 0.83 to 16.9 years, with a mean across all 43 cohorts of 4.2 years. The median number of BMI categories reported was three (interquartile range: 2–4), covering the full underweight to obese range (15.0–35.0 kg/m^2^) (Supplementary Table S2).

The key inputs and outputs for the dose–response meta-analyses are shown in Supplementary Tables S2 and S3. The meta-analyses showed an inverse dose–response relationship between BMI and tuberculosis risk across all studied populations.

**Figure 3.**
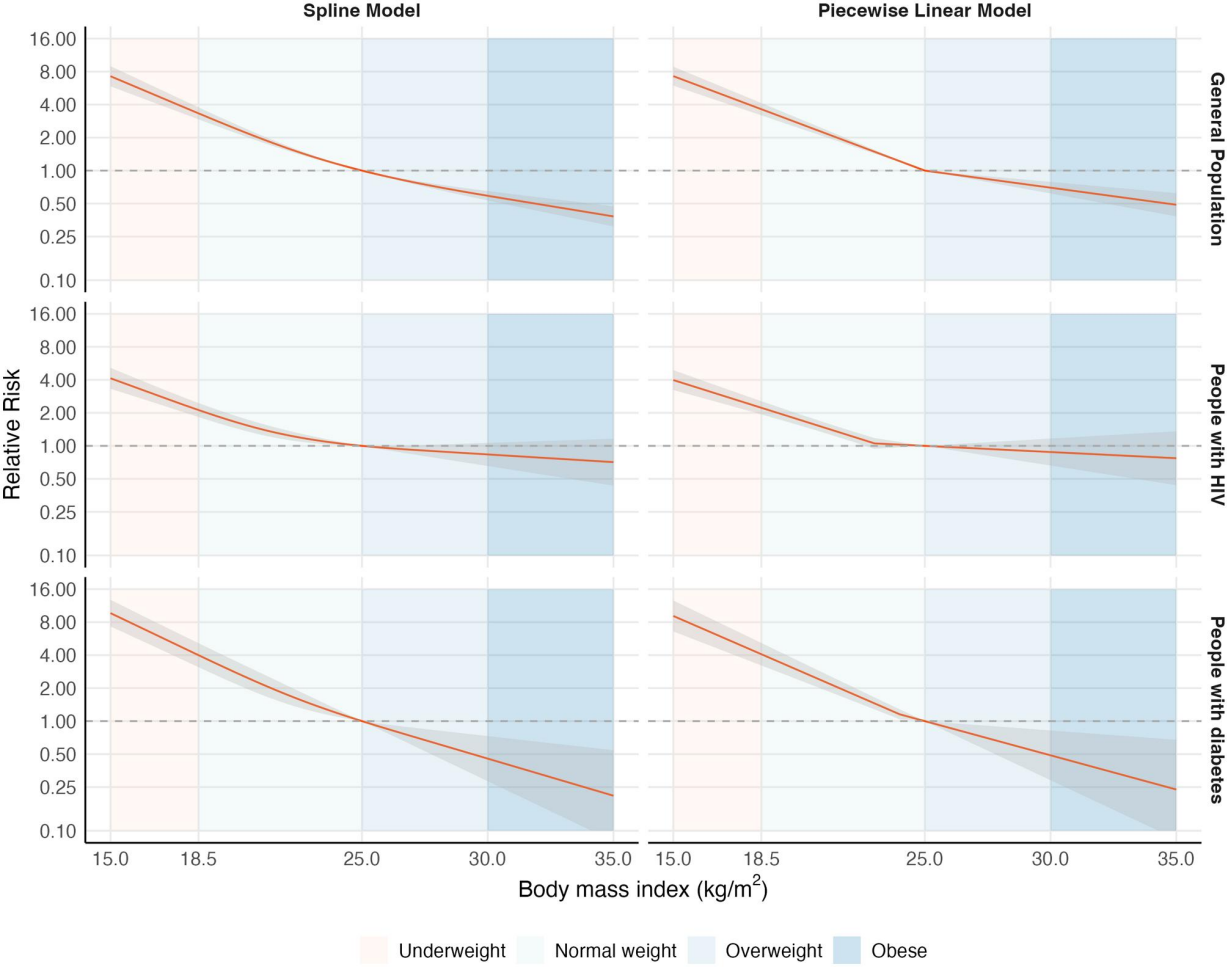
Dose–response meta-analyses fitting restricted cubic spline and piecewise linear models for the relationship between BMI and tuberculosis risk in general-population cohorts; people with HIV; and people with diabetes. General-population cohorts (22 cohorts, *n*=24 921 531); people with HIV (18 cohorts, *n*=162 609); people with diabetes (3 cohorts, *n*=1 118 424). See Table 1 for characteristics of included cohorts and Supplementary Table S3 for the raw data included in the meta-analyses. The solid line represents predictions of RR from the models with reference to a BMI of 25.0 kg/m^2^ and the shaded grey area represents 95% CIs.

The reductions in tuberculosis risk per one-unit increase in BMI for the linear models are shown in Supplementary Figure S1. When fitting spline models, we found evidence for a nonlinear relationship with tuberculosis risk increasing faster at lower BMI values and decreasing more gradually at higher BMI values, particularly among people with HIV (e.g. coefficient for nonlinear term in the general population = 0.12, *P* < 0.001; Supplementary Table S3 and Figure 3). In the piecewise linear models, the reduction in tuberculosis risk per one-unit increase in BMI was 18.0% (95% CI: 16.4–19.6) for BMI of <25.0 kg/m^2^ and 6.9% (95% CI: 4.6–9.2) for BMI of ≥25.0 kg/m^2^ in 22 general-population cohorts (*n* = 24 921 531); 15.3% (95% CI: 13.1–17.5) for BMI of <23.0 kg/m^2^ and 2.6% (95% CI: –3.1–7.9) for BMI of ≥23.0 kg/m^2^ in 18 cohorts of people with HIV (*n* = 162 609); and 20.5% (95% CI: 18.4–22.6) for BMI of <24.0 kg/m^2^ and 13.4% (95% CI: 3.9–22.0) for BMI of ≥24.0 kg/m^2^ in three cohorts of people with diabetes (*n* = 1 118 424) (Figure 3). Similar values were obtained when including only studies performed in countries with a high tuberculosis burden or a lower tuberculosis burden (Supplementary Figure S2).

Across all studied populations, the spline and piecewise linear models had the lowest AIC/BIC values, indicating that they were the best fit for the data (Supplementary Table S3). For all models, the between-study random-effects components were relatively small (e.g. 0.043 for the linear term in the spline model in general-population cohorts), indicating consistency in findings across included studies (Supplementary Table S3).

The RR of tuberculosis associated with undernutrition in different population groups is shown in Table 2. Using the spline model and assuming a mean BMI of 17.0 kg/m^2^ among underweight people globally versus a BMI of 25.6 kg/m^2^ among non-underweight people globally, the RR was 5.0 (95% CI: 4.2–5.9).

**Table 2.** Relative risk of tuberculosis associated with undernutrition (BMI < 18.5 kg/m^2^).

	General population		People with HIV		People with diabetes	
	Spline model RR (95% CI)	Piecewise linear model RR (95% CI)	Spline model RR (95% CI)	Piecewise linear model RR (95% CI)	Spline model RR (95% CI)	Piecewise linear model RR (95% CI)
16.0 vs 25.0 kg/m^2^	5.8 (4.8–7.0)	6.0 (5.0–7.1)	3.4 (2.8–4.1)	3.4 (2.8–4.1)	7.5 (5.7–9.8)	7.2 (5.4–9.8)
Mean BMI among underweight versus non-underweight people globally (17.0 vs 25.6 kg/m^2^)	5.0 (4.2–5.9)	5.1 (4.3–6.0)				
Mean BMI among underweight versus midpoint of normal weight category (17.0 vs 21.8 kg/m^2^)	2.8 (2.5–3.1)	2.6 (2.4–2.9)				

## Discussion

In this updated systematic literature review and dose–response meta-analysis, we refined and extended findings of the 2009 review [7] demonstrating an inverse dose–response relationship between BMI and tuberculosis risk. Through the inclusion of over seven times the number of cohorts and comprehensively accounting for uncertainty in both BMI through resampling and tuberculosis risk through meta-analysis, we confirmed the relationship across the full underweight to obese range (15.0–35.0 kg/m^2^). We now include cohorts from multiple countries with high and lower tuberculosis burden, and demonstrated the relationship not only in general-population cohorts (mostly undertaken in areas with low HIV prevalence), but also in people with HIV and people with diabetes. Importantly, by fitting restricted cubic spline models, we demonstrated that the true relationship between BMI and tuberculosis risk is likely nonlinear and we approximated this with piecewise linear models to provide an interpretable and easily usable percentage reduction in tuberculosis risk per one-unit increase in BMI above and below a breakpoint value. Tuberculosis risk is highest at the lowest BMI values and decreases nonlinearly as BMI increases, with the steepest reduction in risk occurring when moving through underweight and normal weight ranges, and more modest reductions continuing through overweight and obese ranges.

The strength of the dose–response relationship across populations with varying background rates of tuberculosis incidence, even after adjusting for a range of potential confounders, is striking and highlights the fundamental independent importance of nutritional status in tuberculosis epidemiology. This has been recently demonstrated in the RATIONS trial, which showed that nutritional support for household contacts of people with tuberculosis reduced the tuberculosis risk by ∼40% [53]. Importantly, because of the nonlinear relationship, our findings suggest that the protective effects of BMI increases will be most pronounced among the most undernourished populations. We also found that the magnitude of the RR of tuberculosis associated with undernutrition is lower among people with HIV. If undernutrition causes tuberculosis through its effects on cell-mediated immunity [5], then this would be expected given that people with HIV have a competing cause of immune deficiency. Furthermore, we were able to characterize and confirm the dose–response relationship among people with diabetes and show that diabetes is unlikely to be a major contributor to nonlinearity in the general population (estimates for 10 of 22 studies adjusted for diabetes in their multivariable analysis). People with HIV and/or diabetes, who were underrepresented in the RATIONS trial, require specific consideration in future interventional research. Both are more likely to be eligible for tuberculosis preventive treatment, which may modify the effect of nutritional support, and nutritional interventions for people with diabetes need particularly careful design, as their needs and metabolism differ from those of the general population.

Our results should be used to inform WHO calculations of the PAF of tuberculosis due to undernutrition, which previously used a RR estimate informed by the 2009 review [7] of 3.2 when comparing a BMI of 16.0 versus 25.0 kg/m^2^ [6]. The recent Cochrane review, which estimated a RR closer to 2 (HR = 2.2, 95% CI: 1.8–2.7), was undertaken to inform revised PAF calculations but is likely to have substantially underestimated the RR [8]. In its meta-analyses, studies that used different definitions of undernutrition and, more frequently, different comparator groups were inappropriately combined. Given the dose–response relationship between BMI and tuberculosis risk, using different comparator groups is likely to have biased the RR estimate towards 1. Indeed, in several of the studies included in the meta-analyses, the researchers demonstrated the dose–response relationship in their population (including several in which the reference category for the analysis was defined as ‘normal weight’), but these relationships were not considered [13, 19, 20, 22, 24, 29, 47]. Furthermore, for the overall summary estimates, the review pooled together studies performed in the general population in both adults and children with those performed in people with HIV, diabetes, and other rare health conditions (e.g. recent gastrectomy). This may have also biased the RR estimate towards 1 because our results show that the RR associated with undernutrition in people with HIV is likely to be lower, and these accounted for a high relative number of studies in the review’s meta-analyses.

Moving forward, estimates of the PAF of tuberculosis due to undernutrition must account for the dose–response relationship between BMI and tuberculosis risk, and the underlying distribution of BMI in the population. In populations in LMIC where undernutrition is common and tuberculosis burden is higher, the mean BMI among underweight people is likely to be lower than in populations from HIC where undernutrition is rare and the tuberculosis burden is lower. Our dose–response models, stratified by population group, explicitly address these issues and can be used to extract estimates of RR appropriate for different populations.

Further strengths of our review are that we included four studies not published at the time of the Cochrane review [18, 40, 45, 49], several studies for which we were either provided with data or were able to access the original data to calculate required estimates [32, 33, 41, 44, 50], and other important cohorts that were not included in the Cochrane review [21, 25, 26, 30, 31, 34, 39]. This enabled the inclusion of data from >3 million people representing a range of populations/geographies (Supplementary Table S4). Despite this, there remains a lack of evidence in settings with both a high tuberculosis burden and a high prevalence of undernutrition—we found only three small general-population cohorts in the African and South-East Asian Regions.

Our study has other limitations. First, we focussed on adults because of challenges in tuberculosis diagnosis among children and differences in anthropometric measures. Only four studies including children and adolescents were included in the Cochrane review meta-analyses and further research is required in this population by using more standardized measures. Second, our analyses were limited to adjusting for the variables originally adjusted for in each study. No studies explicitly used causal inference methods and only one incorporated sampling weights and survey design specifications, which demonstrated a much higher tuberculosis risk among underweight people [29]. Future studies should state the conceptual framework informing the adjusted analysis and consider methodologies such as propensity score approaches. Finally, despite the clarity of our results and the clinical and practical utility of BMI, research is required to characterize the macro- and micronutritional physiological pathways through which a lower BMI increases risk and, conversely, why a higher BMI decreases risks. Previous research has shown that the protective effect of a high BMI on tuberculosis is much stronger than the harmful effect of a high BMI mediated through an increased risk of diabetes [22]. The mechanisms underpinning this protective effect are poorly understood, but postulated reasons include higher leptin levels in obesity affecting host immunity and adipose tissue acting as a reservoir in which *Mycobacterium tuberculosis* may persist without causing disease [54, 55].

In conclusion, our updated review and dose–response meta-analysis confirms and substantially extends previous research demonstrating the importance of nutritional status for tuberculosis. Given that the historical evidence on improved nutrition and reduced tuberculosis has now been complemented by robust prospective observational, interventional, and modelling evidence, the global tuberculosis community must rapidly ensure that social protection and other interventions to improve nutrition become an integral component of the global tuberculosis response.

## Ethics approval

As this was a review of existing data and involved no primary data collection, ethical approvals were not required for this study.

## Supplementary Material

dyaf154_Supplementary_Data

## Data Availability

All data are incorporated into the article and its online supplementary material.
