# Correction to: Body mass index and tuberculosis risk: an updated systematic literature review and dose–response meta-analysis

**DOI:** 10.1093/ije/dyag010

**Published:** 2026-02-09

This is a correction to: Matthew J Saunders, J Peter Cegielski, Rebecca A Clark, Rein M G J Houben, C Finn McQuaid, Body mass index and tuberculosis risk: an updated systematic literature review and dose–response meta-analysis, *International Journal of Epidemiology*, Volume 54, Issue 5, October 2025, dyaf154, https://doi.org/10.1093/ije/dyaf154

In the originally published version of this manuscript, in the third paragraph of the introduction, the sentence beginning, “Although undernutrition is generally considered to be variable for calculating a PAF” has been corrected to “Although undernutrition is generally considered to be a binary variable for calculating a PAF”.

