# Supplementary material for: Body mass index and tuberculosis risk: an updated systematic literature review and dose–response meta-analysis
Source: Int J Epidemiol. 2025 Sep 8;54(5):dyaf154. doi: 10.1093/ije/dyaf154 (PMC12417079; doi:10.1093/ije/dyaf154)
Supplement: dyaf154_Supplementary_Data [file dyaf154_supplementary_data.docx]

Body mass index and tuberculosis risk –

an updated systematic literature review and dose-response meta-analysis.

**Supplementary material**

**CONTENTS**

| **Item** | **Page** |
| --- | --- |
| Supplementary methods | **1-2** |
| Box S1. Example of optimisation function to predict body mass index distribution | **3** |
| Table S1. Search terms | **4** |
| Table S2. Relative risk of tuberculosis in categories of body mass index in included cohorts and data used in the dose-response meta-analyses. | **5-10** |
| Table S3. Summary of dose-response meta-analysis models for the association between body mass index and tuberculosis | **11** |
| Table S4. Comparison of cohorts included in Franco et al. 2024^1^ meta-analyses and cohorts included in our meta-analyses. | **12-13** |
| Figure S1. Dose-response meta-analyses fitting linear and piecewise linear models for the relationship between body mass index and tuberculosis in a) general population cohorts; b) people with HIV; and c) people with diabetes. | **14** |
| Figure S1. Dose-response meta-analyses fitting linear, piecewise linear, and restricted cubic spline models for the relationship between body mass index and tuberculosis in general population cohorts in high tuberculosis burden countries and lower tuberculosis burden countries. | **15** |
| References for supplementary material | **16-19** |
| PRISMA checklist | **20-23** |

**Supplementary methods**

**Study selection.** Where studies were considered potentially eligible by the two authors (MJS and JPC) screening studies, but the available data were not suitable in their published form, we contacted authors for clarifications and/or to request additional data. Specifically, we requested estimates of tuberculosis incidence in different body mass index (BMI) categories reported as the number of events/number of person-years follow-up; unadjusted and adjusted estimates of the incidence rate ratio (IRR) or hazard ratio (HR) for different BMI categories compared to a reference category; and the mean, standard deviation (SD), and range for BMI in the study population.

Where there were doubts about whether a study should be included, these were resolved in discussion with a third author (CFQ). We checked for overlap of study populations between articles to avoid double-counting. Where there was overlap, we included the article which either already reported data in the correct format; had a primary aim of exploring the relationship between nutritional status and tuberculosis; or had the longest follow-up.

**Handling of BMI.** To select a midpoint BMI “dose” corresponding to each relative risk (RR) for each BMI category to use in the dose-response meta-analysis, we used the median of 1,000 randomly drawn BMI values for each BMI category in each study, assuming that BMI in the overall study population followed a log-normal distribution (e.g. in the 18.0-25.0kg/m^2^ category for a given study, we randomly drew 1,000 values constrained between 18.0 and 25.0). We chose a log-normal distribution as we anticipated that overall BMI would be slightly positively skewed and this distribution was the best fit for BMI for studies where we had access to the raw data. Instead of simply taking the midpoint value in each category (as was done in the 2009 review)^2^, this approach allowed us to appropriately model the uncertainty in the true BMI value within each category while respecting the known distributional properties and boundaries of BMI measurements.

The parameters for the log-normal distribution were calculated from the actual mean and standard deviation (SD) for BMI in the study population. Where these were missing, we estimated them from the median and interquartile range if these were available or used an optimisation function to fit a log-normal distribution to the proportions of study participants in different BMI categories. This function minimised the sum of the squared differences between observed and predicted category quantiles to estimate the distribution parameters. An example is shown in Box S1, illustrating that the estimated distribution parameters closely matched the actual distribution parameters where both were available. In three studies where this not possible (because there were only two BMI categories, and no distributional data were reported) we used a country-estimate of mean BMI from the NCD Risk Factor Collaboration (taking the year estimate closest to when the study was done)^3^ and an SD of four. The method used for each study is shown in Table S2.

For the lowest and highest BMI categories (e.g. ≥30kg/m^2^) where we did not have a minimum or a maximum value, we assigned a minimum value of 10.0kg/m^2^ and a maximum of 50kg/m^2^ based on plausibility and the BMI ranges reported in other studies, except for two older military cohorts of young men where we used 10.0kg/m^2^ and 35.0kg/m^2^ as we did not anticipate obesity class II and III to be present in these two cohorts.

To extract estimates of the mean BMI among underweight (<18.5kg/m^2^) and non-underweight (≥18.5kg/m^2^) people globally, we used the methods described above to fit a log-normal distribution to the proportions of people in different BMI categories reported by the Global Health Observatory.^4^ We then calculated the conditional mean BMIs in the two different groups.

**Box S1.** Example of optimisation function to predict BMI distribution using Aibana et al. 2016^5^

**Actual BMI distribution reproduced from raw data**

Mean=26.6kg/m^2^; SD=4.77kg/m^2^


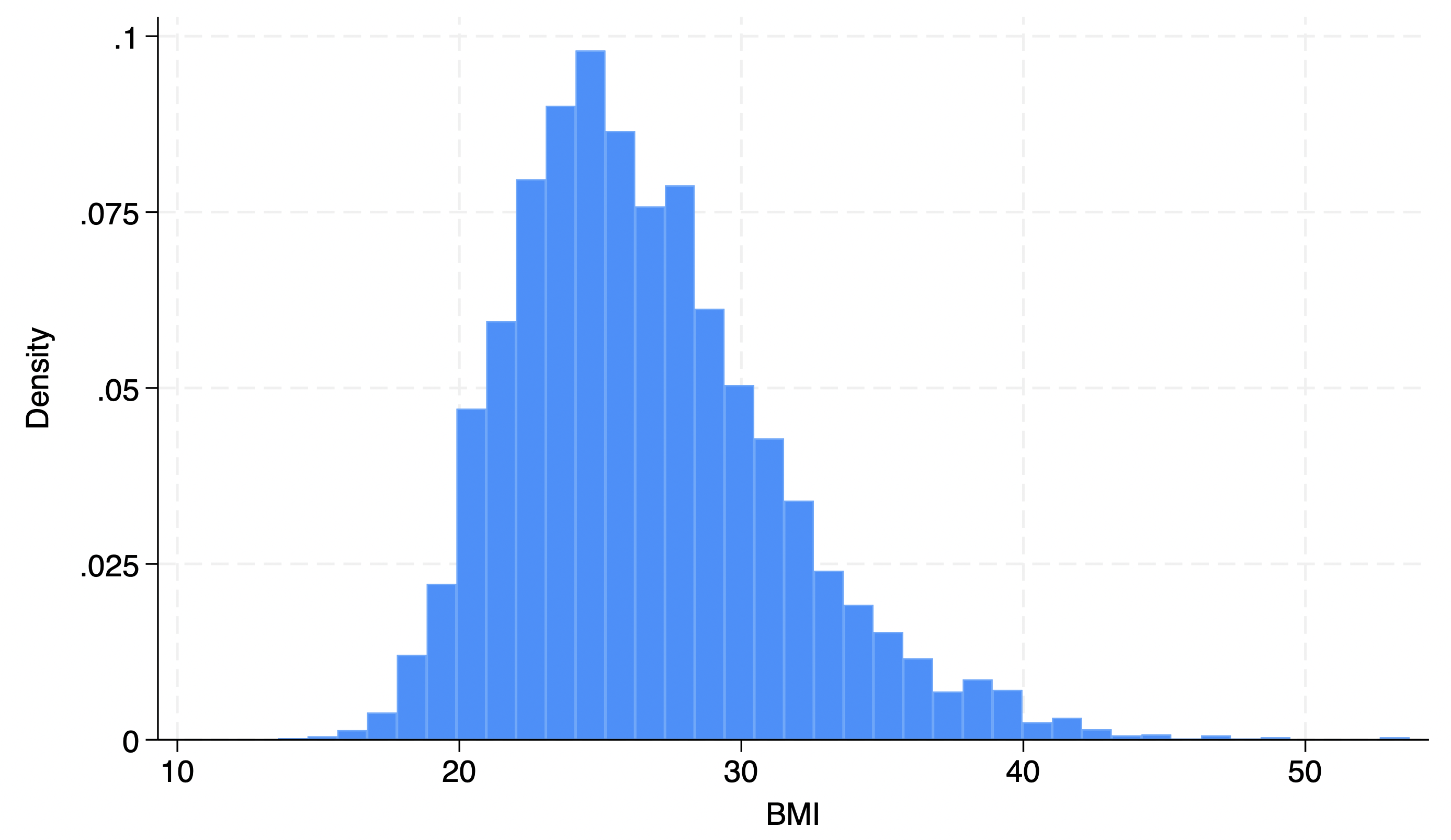


**Fitted distribution using optimisation function from four derived BMI groups.**

Estimated mean=26.4kg/m^2^; Estimated SD=4.4kg/m^2^

| *BMI category* | *Number in category (n=7,606)* | *Proportion in category* |
| --- | --- | --- |
| <20.0kg/m^2^ | 360 | 4.7% |
| 20-24.9kg/m^2^ | 2,853 | 37.5% |
| 25.0-29.9kg/m^2^ | 2,783 | 36.6% |
| ≥30.0kg/m^2^ | 1,610 | 21.2% |


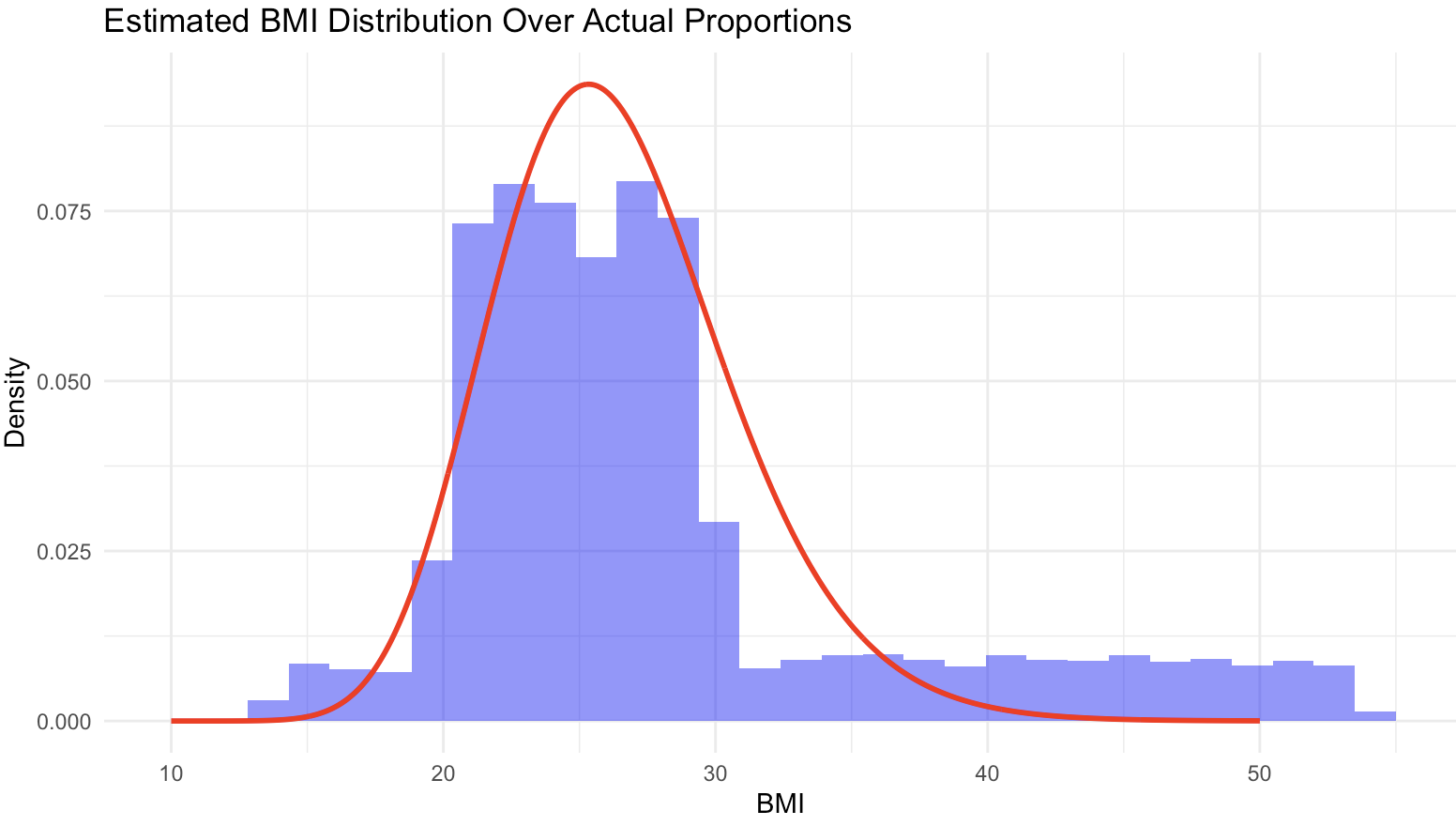


Note. The histogram represents uniformly randomly sampled values in the different BMI categories

**Table S1.** Search terms

| **Ovid MEDLINE(R) ALL <1946 to March 28, 2024>** |
| --- |
| 1 exp tuberculosis/ 208800  2 mycobacterium tuberculosis/ 58652  3 (tuberculosis or tb).ti,ab. 245652  4 or/1-3 307538  5 Malnutrition/ 20445  6 Thinness/ 8094  7 Wasting Syndrome/ 1468  8 Nutrition Disorders/ 18325  9 body weight/ or weight loss/ 241972  10 body mass index/ 151937  11 (undernutr* or malnutr* or underweight or nutri* or weight or wasting or thin or thinness or "body mass index" or bmi).ti,ab. 1905099  12 or/5-11 2031904  13 epidemiologic studies/ or cohort studies/ or prospective studies/ 977163  14 (prospective or inciden* or risk or rate*).ti,ab. 6800263  15 or/13-14 7061957  16 4 and 12 and 15 4551  17 exp animals/ not humans.sh. 5207360  18 16 not 17 4331  19 limit 18 to yr="2008 -Current" 3220 |
| **Embase <1980 to 2024 Week 12>** |
| 1 exp tuberculosis/ 215545  2 mycobacterium tuberculosis/ 74725  3 (tuberculosis or tb).ti,ab. 234458  4 or/1-3 299694  5 Malnutrition/ 77391  6 Underweight/ 24300  7 Wasting Syndrome/ 5054  8 Nutritional Disorders/ 0  9 body weight/ or body weight loss/ 464959  10 body mass/ 667658  11 (undernutr* or malnutr* or underweight or nutri* or weight or wasting or thin or thinness or "body mass index" or bmi).ti,ab. 2479478  12 or/5-11 2788849  13 epidemiology/ or cohort analysis/ or prospective study/ 2051296  14 (prospective or inciden* or risk or rate*).ti,ab. 9226943  15 or/13-14 9856375  16 4 and 12 and 15 9890  17 exp animal/ not human.sh. 4894520  18 16 not 17 9543  19 limit 18 to yr="2008 -Current" 8423 |

**Table S2.** Relative risk of tuberculosis in categories of body mass index (BMI) in included cohorts and data used in the dose-response meta-analyses.

| **Author** | **BMI in study population** | | | **BMI categories** | | | **Relative risk data** | | | | | | **Notes** |
| --- | --- | --- | --- | --- | --- | --- | --- | --- | --- | --- | --- | --- | --- |
|  | **Mean BMI in study population** | **SD for BMI in study population** | **Method for estimating mean/SD** | **BMI low** | **BMI high** | **Median re-sampled BMI** | **No of cases** | **No of person-years** | **IRR or HR** | **IRR** | **IRR low** | **IRR high** |  |
| Palmer et al. 1957^6^ | 21.9 | 2.27 | Optimisation function | 10.0 | 19.2 | 18.4 | 22 | 29290 | IRR | 3.98 | 1.85 | 9.09 | IRRs calculated from raw data |
|  |  |  |  | 19.3 | 20.4 | 19.9 | 29 | 43076 | IRR | 3.57 | 1.73 | 7.91 |  |
|  |  |  |  | 20.5 | 21.5 | 21.0 | 20 | 56749 | IRR | 1.87 | 0.85 | 4.31 |  |
|  |  |  |  | 21.6 | 23.7 | 22.6 | 26 | 87634 | IRR | 1.57 | 0.75 | 3.52 |  |
|  |  |  |  | 23.8 | 35.0 | 24.9 | 11 | 58260 | IRR | Ref | Ref | Ref |  |
| Edwards et al. 1971^7^ | 22.1 | 2.71 | Optimisation function | 10.0 | 19.6 | 18.6 | 115 | 581328 | IRR | 3.52 | 2.47 | 5.1 | IRRs calculated from raw data and person-years approximated |
|  |  |  |  | 19.7 | 23.9 | 21.8 | 224 | 1928016 | IRR | 2.07 | 1.49 | 2.93 |  |
|  |  |  |  | 24.0 | 35.0 | 25.5 | 44 | 783492 | IRR | Ref | Ref | Ref |  |
| Hemila et al. 1999^8^ | 26.3 | 3.81 | Real | 13.0 | 22.9 | 21.6 | 63 | 32873 | HR | Ref | Ref | Ref | Person-years calculated from case and rate data |
|  |  |  |  | 23.0 | 27.0 | 25.1 | 67 | 72666 | HR | 0.48 | 0.34 | 0.68 |  |
|  |  |  |  | 27.1 | 54.4 | 29.5 | 37 | 67476 | HR | 0.29 | 0.19 | 0.44 |  |
| Tverdal 1986^9^ | 26.3 | 3.81 | Optimisation function | 16.4 | 20.9 | 20.0 | 494 | 3446742 | IRR | 5.39 | 3.86 | 7.51 | IRRs calculated from incidence rates reported in previous review^2^ as age/sex stratified and original publication only provides estimates disaggregated by sex alongside raw data. |
|  |  |  |  | 21.0 | 22.9 | 22.1 | 566 | 4432261 | IRR | 3.61 | 2.59 | 5.04 |  |
|  |  |  |  | 23.0 | 24.9 | 24.1 | 579 | 4589834 | IRR | 2.68 | 1.91 | 3.76 |  |
|  |  |  |  | 25.0 | 26.9 | 25.9 | 398 | 3528653 | IRR | 2.00 | 1.41 | 2.83 |  |
|  |  |  |  | 27.0 | 28.9 | 27.9 | 255 | 2213079 | IRR | 1.32 | 0.91 | 1.93 |  |
|  |  |  |  | 29.0 | 30.9 | 29.9 | 122 | 1236696 | IRR | 0.9 | 0.56 | 1.46 |  |
|  |  |  |  | 31.0 | 44.9 | 32.8 | 117 | 1279437 | IRR | Ref | Ref | Ref |  |
| Leung et al. 2007^10^ | 24.2 | 3.63 | Optimisation function | 10.0 | 18.4 | 17.6 | 69 | 9426 | HR | 2.11 | 1.59 | 2.79 | Person-years calculated from case and rate data |
|  |  |  |  | 18.5 | 22.9 | 21.3 | 191 | 65636 | HR | Ref | Ref | Ref |  |
|  |  |  |  | 23.0 | 24.9 | 23.9 | 97 | 48500 | HR | 0.74 | 0.58 | 0.94 |  |
|  |  |  |  | 25.0 | 29.9 | 26.8 | 109 | 73649 | HR | 0.58 | 0.46 | 0.74 |  |
|  |  |  |  | 30.0 | 50.0 | 31.7 | 11 | 13415 | HR | 0.38 | 0.21 | 0.7 |  |
| Cegielski et al. 2012^11^ | 25.6 | 5.15 | Real | 12.6 | 18.4 | 17.3 | 11 | 6682 | HR | 12.43 | 5.75 | 26.95 |  |
|  |  |  |  | 18.5 | 24.9 | 22.3 | 32 | 102498 | HR | Ref | Ref | Ref |  |
|  |  |  |  | 25.0 | 29.9 | 27.1 | 14 | 71054 | HR | 0.28 | 0.13 | 0.63 |  |
|  |  |  |  | 30.0 | 72.3 | 32.7 | 4 | 35288 | HR | 0.20 | 0.07 | 0.62 |  |
| Cho et al. 2022^12^ | 23.6 | 3.15 | Optimisation function | 10.0 | 15.9 | 15.5 | 357 | 107851 | HR | 2.83 | 2.55 | 3.15 |  |
|  |  |  |  | 16.0 | 16.9 | 16.6 | 786 | 376382 | HR | 2.50 | 2.33 | 2.68 |  |
|  |  |  |  | 17.0 | 18.4 | 17.9 | 3406 | 2173728 | HR | 1.98 | 1.91 | 2.05 |  |
|  |  |  |  | 18.5 | 22.9 | 21.1 | 26710 | 27637461 | HR | Ref | Ref | Ref |  |
|  |  |  |  | 23.0 | 24.9 | 23.9 | 10976 | 17321680 | HR | 0.56 | 0.55 | 0.58 |  |
|  |  |  |  | 25.0 | 50.0 | 26.8 | 10380 | 22446051 | HR | 0.40 | 0.39 | 0.41 |  |
| Lin et al. 2018^13^ NHIS | 22.5 | 4.18 | Real | 10.0 | 18.4 | 17.1 | 20 | 24168 | HR | 2.28 | 1.41 | 3.69 |  |
|  |  |  |  | 18.5 | 24.9 | 21.7 | 118 | 242725 | HR | Ref | Ref | Ref |  |
|  |  |  |  | 25.0 | 29.9 | 26.7 | 28 | 93130 | HR | 0.48 | 0.32 | 0.73 |  |
|  |  |  |  | 30.0 | 49.2 | 31.7 | 3 | 19091 | HR | 0.33 | 0.1 | 1.03 |  |
| Lin et al. 2018^13^ NTC | 24.4 | 3.61 | Real | 12.5 | 18.4 | 17.6 | 22 | 23831 | HR | 2.44 | 1.57 | 3.8 |  |
|  |  |  |  | 18.5 | 24.9 | 22.4 | 211 | 505089 | HR | Ref | Ref | Ref |  |
|  |  |  |  | 25.0 | 29.9 | 26.7 | 79 | 280504 | HR | 0.53 | 0.41 | 0.68 |  |
|  |  |  |  | 30.0 | 48.9 | 31.5 | 10 | 59217 | HR | 0.36 | 0.19 | 0.69 |  |
| Chen et al. 2022^14^ | 22.6 | 3.20 | Real | 10.4 | 18.4 | 17.6 | 6 | 3465 | HR | 1.25 | 0.52 | 3.04 |  |
|  |  |  |  | 18.5 | 23.9 | 21.5 | 31 | 27382 | HR | Ref | Ref | Ref |  |
|  |  |  |  | 24.0 | 69.9 | 25.8 | 6 | 13728 | HR | 0.34 | 0.14 | 0.83 |  |
| Cheng et al. 2020^15^ | 22.4 | 3.77 | Real | 11.4 | 18.4 | 17.2 | 17 | 4789 | HR | 2.33 | 1.32 | 4.12 |  |
|  |  |  |  | 18.5 | 23.9 | 21.4 | 39 | 27380 | HR | Ref | Ref | Ref |  |
|  |  |  |  | 24.0 | 57.9 | 26.1 | 6 | 12445 | HR | 0.34 | 0.14 | 0.8 |  |
| Kim et al. 2018^16^ | 23.5 | 3.15 | Optimisation function | 10.0 | 18.4 | 17.7 | 257 | 73646 | HR | 1.36 | 1.16 | 1.61 |  |
|  |  |  |  | 18.5 | 22.9 | 21.2 | 1893 | 757522 | HR | Ref | Ref | Ref |  |
|  |  |  |  | 23.0 | 24.9 | 24.0 | 839 | 477851 | HR | 0.63 | 0.57 | 0.69 |  |
|  |  |  |  | 25.0 | 29.9 | 26.6 | 720 | 551784 | HR | 0.45 | 0.4 | 0.5 |  |
|  |  |  |  | 30.0 | 50.0 | 31.2 | 63 | 58303 | HR | 0.4 | 0.3 | 0.54 |  |
| Choi et al. 2021^17^ (no diabetes) | 23.6 | 3.17 | Real | 10.0 | 18.4 | 17.6 | 4725 | 2632033 | HR | 2.21 | 2.14 | 2.28 |  |
|  |  |  |  | 18.5 | 22.9 | 21.2 | 24902 | 27196588 | HR | Ref | Ref | Ref |  |
|  |  |  |  | 23.0 | 24.9 | 23.9 | 9169 | 16592920 | HR | 0.54 | 0.53 | 0.55 |  |
|  |  |  |  | 25.0 | 29.9 | 26.5 | 7004 | 18469674 | HR | 0.36 | 0.35 | 0.37 |  |
|  |  |  |  | 30.0 | 50.0 | 31.2 | 558 | 2091306 | HR | 0.28 | 0.26 | 0.31 |  |
| Jiang et al. 2024^18^ | 24.1 | 3.16 | Estimated from median/IQR | 10.0 | 18.4 | 17.7 | 40 | 10249 | HR | 9.89 | 4.92 | 19.85 |  |
|  |  |  |  | 18.5 | 23.9 | 22.0 | 136 | 126773 | HR | 2.87 | 1.51 | 5.46 |  |
|  |  |  |  | 24.0 | 27.9 | 25.6 | 60 | 100407 | HR | 1.64 | 0.84 | 3.21 |  |
|  |  |  |  | 28.0 | 50.0 | 29.4 | 10 | 29352 | HR | Ref | Ref | Ref |  |
| Soh et al. 2019^19^ | 23.2 | 3.57 | Real | 9.8 | 18.4 | 17.5 | 164 | 57949 | HR | Ref | Ref | Ref |  |
|  |  |  |  | 18.5 | 24.9 | 22.1 | 640 | 568593 | HR | 0.46 | 0.39 | 0.55 |  |
|  |  |  |  | 25.0 | 29.9 | 26.6 | 162 | 195395 | HR | 0.35 | 0.28 | 0.44 |  |
|  |  |  |  | 30.0 | 69.0 | 31.3 | 24 | 31735 | HR | 0.36 | 0.23 | 0.55 |  |
| Yoo et al. 2021^20^ | 24.3 | 3.00 | Real | 10.0 | 18.4 | 17.8 | 648 | 151232 | HR | 2.36 | 2.17 | 2.57 |  |
|  |  |  |  | 18.5 | 22.9 | 21.5 | 4203 | 2549409 | HR | Ref | Ref | Ref |  |
|  |  |  |  | 23.0 | 24.9 | 24.0 | 2237 | 2286235 | HR | 0.6 | 0.57 | 0.64 |  |
|  |  |  |  | 25.0 | 29.9 | 26.6 | 2105 | 2877801 | HR | 0.45 | 0.43 | 0.48 |  |
|  |  |  |  | 30.0 | 50.0 | 31.2 | 151 | 290267 | HR | 0.33 | 0.28 | 0.39 |  |
| Saunders et al. 2017^21^ | 25.4 | 4.25 | Real | 15.6 | 19.9 | 18.8 | 12 | 356 | HR | 1.58 | 0.83 | 3.01 |  |
|  |  |  |  | 20.0 | 24.9 | 22.8 | 46 | 2060 | HR | Ref | Ref | Ref |  |
|  |  |  |  | 25.0 | 29.9 | 27.1 | 20 | 1785 | HR | 0.52 | 0.3 | 0.9 |  |
|  |  |  |  | 30.0 | 46.4 | 31.8 | 5 | 669 | HR | 0.34 | 0.13 | 0.87 |  |
| Aibana et al. 2016^5^ | 26.6 | 4.77 | Real | 13.6 | 19.9 | 18.7 | 27 | 330 | HR | 2.12 | 1.38 | 3.25 |  |
|  |  |  |  | 20.0 | 24.9 | 23.0 | 126 | 2746 | HR | Ref | Ref | Ref |  |
|  |  |  |  | 25.0 | 29.9 | 27.2 | 56 | 2710 | HR | 0.5 | 0.36 | 0.7 |  |
|  |  |  |  | 30.0 | 53.7 | 32.6 | 25 | 1564 | HR | 0.4 | 0.25 | 0.63 |  |
| Saunders et al. 2020^22^ | 25.6 | 3.94 | Real | 11.3 | 18.4 | 17.6 | 21 | 533 | HR | 2.33 | 1.49 | 3.64 |  |
|  |  |  |  | 18.5 | 24.9 | 22.8 | 253 | 15387 | HR | Ref | Ref | Ref |  |
|  |  |  |  | 25 | 29.9 | 27.0 | 98 | 12331 | HR | 0.52 | 0.41 | 0.66 |  |
|  |  |  |  | 30 | 47.9 | 31.8 | 22 | 4018 | HR | 0.37 | 0.23 | 0.58 |  |
| Larrson et al. 2025^23^ | 22.5 | 5.57 | Real | 10.8 | 18.5 | 16.5 | 13 | 560 | HR | 1.69 | 0.74 | 3.88 |  |
|  |  |  |  | 18.5 | 24.9 | 21.6 | 19 | 1595 | HR | Ref | Ref | Ref |  |
|  |  |  |  | 25 | 54.5 | 28.0 | 6 | 942 | HR | 0.38 | 0.14 | 1 |  |
| Paradakar et al. 2020^24^ | 23.5 | 5.03 | Real | 9.9 | 18.4 | 16.9 | 5 | 169 | IRR | 9.88 | 2.07 | 47.1 |  |
|  |  |  |  | 18.5 | 46 | 24.1 | 9 | 883 | IRR | Ref | Ref | Ref |  |
| Sinha et al. 2024^25^ | 22.3 | 5.48 | Real | 10.8 | 18.4 | 16.5 | 10 | 521 | HR | 1.22 | 0.42 | 3.57 |  |
|  |  |  |  | 18.5 | 22.9 | 20.8 | 7 | 538 | HR | Ref | Ref | Ref |  |
|  |  |  |  | 23 | 48.5 | 26.3 | 1 | 791 | HR | 0.11 | 0.01 | 0.96 |  |
| Batista et al. 2013^26^ | 23.7 | 4.23 | Real | 12.7 | 18.4 | 17.2 | 25 | 337 | HR | 2.27 | 1.46 | 3.53 |  |
|  |  |  |  | 18.5 | 24.9 | 22.0 | 95 | 3031 | HR | Ref | Ref | Ref |  |
|  |  |  |  | 25 | 29.9 | 26.9 | 24 | 1399 | HR | 0.56 | 0.36 | 0.88 |  |
|  |  |  |  | 30 | 44.8 | 32.0 | 1 | 414 | HR | 0.079 | 0.011 | 0.57 |  |
| Tchakounte Youngui et al. 2020^27^ | 21.3 | 4.36 | Real | 10.5 | 16 | 14.8 | 22 | 308 | IRR | 4.77 | 2.47 | 9.17 |  |
|  |  |  |  | 16.1 | 20.9 | 18.9 | 65 | 2167 | IRR | 1.99 | 1.21 | 3.36 |  |
|  |  |  |  | 21 | 62.5 | 24.2 | 30 | 2317 | IRR | Ref | Ref | Ref |  |
| Kyaw et al. 2022^28^ | 20.5 | 4.76 | Real | 8.8 | 18.4 | 16.3 | 1901 | 16801 | HR | 1.97 | 1.83 | 2.11 |  |
|  |  |  |  | 18.5 | 22.9 | 20.4 | 1206 | 23980 | HR | Ref | Ref | Ref |  |
|  |  |  |  | 23 | 27.5 | 24.7 | 319 | 8878 | HR | 0.74 | 0.65 | 0.84 |  |
|  |  |  |  | 27.6 | 45 | 29.8 | 202 | 4223 | HR | 0.93 | 0.8 | 1.08 |  |
| Maro et al. 2010^29^ | 24.5 | 4.75 | Real | 16.1 | 18.4 | 17.6 | 7 | 179 | IRR | 1.31 | 0.51 | 2.82 | IRRs calculated from raw data |
|  |  |  |  | 18.5 | 47.4 | 24.7 | 85 | 2849 | IRR | Ref | Ref | Ref |  |
| Maokola et al. 2021^30^ | 22.9 | 4.84 | Optimisation function | 10 | 18.4 | 16.9 | 1635 | 17275 | HR | 1.73 | 1.62 | 1.84 |  |
|  |  |  |  | 18.5 | 24.9 | 21.8 | 2692 | 73566 | HR | Ref | Ref | Ref |  |
|  |  |  |  | 25 | 29.9 | 26.9 | 458 | 25022 | HR | 0.69 | 0.63 | 0.76 |  |
|  |  |  |  | 30 | 50 | 32.3 | 143 | 12462 | HR | 0.48 | 0.4 | 0.56 |  |
| Salvadori et al. 2015^31^ | 21.3 | 3.64 | Real | 11.2 | 18.4 | 17.0 | 41 | 2052 | HR | 2.9 | 1.9 | 4.3 |  |
|  |  |  |  | 18.5 | 41.9 | 22.0 | 46 | 8171 | HR | Ref | Ref | Ref |  |
| Chang et al. 2015^32^ | 22.2 | 4.15 | Real | 9.8 | 15.9 | 15.0 | 68 | 394 | HR | 4.12 | 2.2 | 7.72 | Time-updated BMI estimates used |
|  |  |  |  | 16 | 18.4 | 17.4 | 183 | 2604 | HR | 2.14 | 1.62 | 2.81 |  |
|  |  |  |  | 18.5 | 24.9 | 21.5 | 877 | 31909 | HR | Ref | Ref | Ref |  |
|  |  |  |  | 25 | 29.9 | 26.7 | 283 | 15552 | HR | 0.78 | 0.67 | 0.92 |  |
|  |  |  |  | 30 | 58 | 31.7 | 91 | 6390 | HR | 0.46 | 0.23 | 0.93 |  |
| Nguenha et al. 2025^33^ | 24.7 | 5.46 | Estimated from median/IQR | 10 | 18.5 | 17.1 | 15 | 457 | HR | 2.6 | 1.4 | 4.8 |  |
|  |  |  |  | 18.5 | 24.9 | 21.8 | 39 | 3000 | HR | Ref | Ref | Ref |  |
|  |  |  |  | 25 | 29.9 | 27.2 | 13 | 1500 | HR | 0.6 | 0.3 | 1.1 |  |
|  |  |  |  | 30 | 50 | 32.6 | 9 | 1100 | HR | 0.5 | 0.2 | 1 |  |
| Mupfumi et al. 2018^34^ | 22.2 | 6.11 | Real | 13.8 | 18.4 | 16.6 | 7 | 15685 | HR | 6.57 | 1.95 | 22.1 |  |
|  |  |  |  | 18.5 | 61.9 | 23.9 | 5 | 131869 | HR | Ref | Ref | Ref |  |
| Alemu et al. 2020^35^ | 20.7 | 3.76 | Real | 13 | 18.4 | 16.9 | 72 | 532 | HR | 1.91 | 1.53 | 2.69 |  |
|  |  |  |  | 18.5 | 36.1 | 21.8 | 74 | 1608 | HR | Ref | Ref | Ref |  |
| Tiruneh et al. 2018^36^ | 20.6 | 4.00 | NCD risk factor data* | 10 | 18.4 | 16.6 | 18 | 903 | HR | 1.85 | 1.02 | 3.55 |  |
|  |  |  |  | 18.5 | 50 | 21.9 | 35 | 579 | HR | Ref | Ref | Ref |  |
| Ahmed et al. 2018^37^ | 18.3 | 2.49 | Real | 9.6 | 18.4 | 16.7 | 75 | 626 | HR | 2.53 | 1.27 | 5.05 |  |
|  |  |  |  | 18.5 | 29.1 | 20.0 | 42 | 751 | HR | Ref | Ref | Ref |  |
| Aaemro et al. 2020^38^ | 20.9 | 3.58 | Estimated from median/IQR | 10 | 18.4 | 16.8 | 47 | 288 | HR | 1.97 | 0.99 | 3.88 |  |
|  |  |  |  | 18.5 | 50 | 21.7 | 15 | 713 | HR | Ref | Ref | Ref |  |
| Nicholas et al. 2011^39^ (during ART) | 19.9 | 3.10 | Estimated from median/IQR | 10 | 18.4 | 16.9 | 436 | 5260 | IRR | 1.96 | 1.69 | 2.22 |  |
|  |  |  |  | 18.5 | 50 | 21.1 | 465 | 11249 | IRR | Ref | Ref | Ref |  |
| Nicholas et al. 2011^39^ (pre-ART) | 20.5 | 2.85 | Estimated from median/IQR | 10 | 18.4 | 17.2 | 362 | 1536 | IRR | 2.78 | 2.38 | 3.22 |  |
|  |  |  |  | 18.5 | 50 | 21.3 | 406 | 5777 | IRR | Ref | Ref | Ref |  |
| Kufa et al. 2016^40^ | 25.1 | 5.47 | Real | 14.5 | 18.4 | 17.2 | 3 | 32 | HR | 3.87 | 1.09 | 13.73 |  |
|  |  |  |  | 18.5 | 57.4 | 24.8 | 12 | 521 | HR | Ref | Ref | Ref |  |
| Moore et al. 2007^41^ | 21.5 | 4.00 | NCD risk factor data* | 10 | 18 | 16.7 | 26 | 325 | HR | 2.8 | 1.59 | 4.92 | Person-years calculated from rate data |
|  |  |  |  | 18.1 | 50 | 22.1 | 26 | 1034 | HR | Ref | Ref | Ref |  |
| Choun et al. 2013^42^ | 19.5 | 3.57 | Optimisation function | 10 | 18.4 | 16.4 | 96 | 510 | HR | 1.6 | 1.1 | 2.2 |  |
|  |  |  |  | 18.5 | 50 | 21.2 | 67 | 770 | HR | Ref | Ref | Ref |  |
| Choi et al. 2021^17^ (diabetes) | 25.0 | 3.27 | Real | 10 | 18.4 | 17.8 | 448 | 83771 | HR | 2.13 | 1.93 | 2.35 |  |
|  |  |  |  | 18.5 | 22.9 | 21.7 | 3532 | 1566537 | HR | Ref | Ref | Ref |  |
|  |  |  |  | 23 | 24.9 | 24.0 | 2040 | 1619120 | HR | 0.58 | 0.55 | 0.61 |  |
|  |  |  |  | 25 | 29.9 | 26.9 | 2308 | 2569395 | HR | 0.44 | 0.41 | 0.46 |  |
|  |  |  |  | 30 | 50 | 31.3 | 218 | 438279 | HR | 0.29 | 0.25 | 0.33 |  |
| Li et al. 2020^43^ | 24.2 | 3.00 | Optimisation function | 10 | 18.4 | 17.8 | 27 | 12548 | IRR | 3.33 | 2.15 | 4.93 | IRRs calculated from raw data |
|  |  |  |  | 18.5 | 24.9 | 22.7 | 321 | 496125 | IRR | Ref | Ref | Ref |  |
|  |  |  |  | 25 | 50 | 26.9 | 85 | 329073 | IRR | 0.4 | 0.31 | 0.51 |  |
| Gedfew et al. 2020^44^ | 20.6 | 4.00 | NCD risk factor data* | 10 | 18.4 | 16.7 | 13 | 213 | HR | 2.94 | 1.25 | 7.14 |  |
|  |  |  |  | 18.5 | 50 | 21.9 | 13 | 889 | HR | Ref | Ref | Ref |  |

ART, Antiretroviral therapy; HR, Hazard ratio; IRR, Incidence rate ratio; IQR, Interquartile range; NHIS, National Health Interview Surveys; NTC, New Taipei City; SD, Standard deviation

*NCD risk factor data.^3^

Saunders et al. 2017 and 2020,^21,22^ Maokola. et al 2021,^30^ Aibana et al. 2016,^5^ and Batista et al. 2013^26^ were re-analyses of datasets that were either available to the authors, publicly available, or provided by the original study authors. All other data were either directly extracted from published reports, calculated from raw data presented in published reports, or provided to the authors by the original study authors.

**Table S3.** Summary of dose-response meta-analysis models for the association between body mass index and tuberculosis risk.

| **Model type** | | **Coefficient (95%CI)** | **p value** | **AIC** | **BIC** | **Between-study random-effects components (standard deviation)** |
| --- | --- | --- | --- | --- | --- | --- |
| **General population. 22 cohorts (n=24,921,531)** | | | | | | |
| Linear | Effect | -0.1607 (-0.1783, -0.1432) | <0.001 | 677.7 | 682.1 | 0.0330 |
| Spline model  Knots at: 17.6, 22.9, 31.2 | Linear term | -0.2230 (-0.2483, -0.1976) | <0.001 | 155.3 | 166.2 | 0.0425 |
|  | Non-linear term | 0.1161 (0.0872, 0.1451) | <0.001 |  |  | 0.0364 |
| Piecewise linear model  Breakpoint:  25.0 | Effect below breakpoint | -0.1984 (-0.2183, -0.1786) | <0.001 | 24.9 | 35.8 | 0.0345 |
|  | Change in slope above breakpoint | 0.1266 (0.0977, 0.1555) | <0.001 |  |  | 0.0360 |
| **General population, high tuberculosis burden countries. 9 cohorts (n=123,809)** | | | | | | |
| Linear | Effect | -0.1561 (-0.1855, -0.1267) | <0.001 | 28.0 | 30.0 | 0.0254 |
| Spline model  Knots at: 17.2, 22.8, 29.7 | Linear term | -0.2173 (-0.2759, -0.1588) | <0.001 | 30.0 | 34.8 | 0.0412 |
|  | Non-linear term | 0.0857 (0.0216, 0.1497) | 0.009 |  |  | 0.0396 |
| Piecewise linear model  Breakpoint:  25.0 | Effect below breakpoint | -0.2025 (-0.2365, -0.1684) | <0.001 | 27.7 | 32.4 | <0.001 |
|  | Change in slope above breakpoint | 0.1233 (0.0564, 0.1903) | <0.001 |  |  | <0.001 |
| **General population, lower tuberculosis burden countries. 13 cohorts (n=24,797,722)** | | | | | | |
| Linear | Effect | -0.1626 (-0.1851, -0.1401) | <0.001 | 659.3 | 662.9 | 0.0368 |
| Spline model  Knots at: 17.6, 23.9, 31.2 | Linear term | -0.2243 (-0.2564, -0.1921) | <0.001 | 112.5 | 121.6 | 0.0498 |
|  | Non-linear term | 0.1057 (0.0775, 0.1339) | <0.001 |  |  | 0.0337 |
| Piecewise linear model  Breakpoint:  25.0 | Effect below breakpoint | -0.1997 (-0.2265, -0.1730) | <0.001 | 14.1 | 23.1 | 0.0426 |
|  | Change in slope above breakpoint | 0.1298 (0.0962, 0.1634) | <0.001 |  |  | 0.0405 |
| **People with HIV. 18 cohorts (n=162,609)** | | | | | | |
| Linear | Effect | -0.1408 (-0.1693, -0.1123) | <0.001 | 187.7 | 190.5 | 0.0481 |
| Spline model  Knots at: 16.6; 21.3; 28.0 | Linear term | -0.1924 (-0.2274, -0.1573) | <0.001 | 30.0 | 36.7 | 0.0497 |
|  | Non-linear term | 0.1304 (0.0712, 0.1896) | <0.001 |  |  | 0.0681 |
| Piecewise linear model  Breakpoint: 23.0 | Effect below breakpoint | -0.1663 (-0.1926, -0.1400) | <0.001 | 29.9 | 36.5 | 0.0386 |
|  | Change in slope above breakpoint | 0.1404 (0.0715, 0.2092) | <0.001 |  |  | 0.0828 |
| **People with diabetes. 3 cohorts (n=1,118,424)** | | | | | | |
| Linear | Effect | -0.1907 (-0.2478, -0.1335) | <0.001 | 91.3 | 90.9 | 0.0404 |
| Spline model  Knots at: 17.7; 22.3; 27.3 | Linear term | -0.2525 (-0.2777, -0.2272) | <0.001 | 45.5 | 43.6 | 0.0023 |
|  | Non-linear term | 0.0676 (-0.0059, 0.1410) | 0.0716 |  |  | 0.0472 |
| Piecewise linear model  Breakpoint:  24.0 | Effect below breakpoint | -0.2295 (-0.2557, -0.2033) | <0.001 | 12.4 | 10.4 | 0.0152 |
|  | Change in slope above breakpoint | 0.0858 (0.0007, 0.1710) | 0.0482 |  |  | 0.0558 |

95%CI; 95% confidence interval; AIC; Akaike Information Criterion; BIC; Bayesian Information Criterion; BMI; Body mass index; RR; Relative risk

**Table S4.** Comparison of cohorts included in Franco et al. 2024^1^ meta-analyses and cohorts included in our meta-analyses.

| **Study/Cohort** | **Number of people** | **Population** | **Reason for difference** |
| --- | --- | --- | --- |
| **Cohorts included in Franco et al. 2024^1^ meta-analyses not included in our meta-analyses (additional participants excluding overlap and wrong populations = 1,522,868)** | | | |
| Baker et al. 2012^45^ | 17,715 | General population | Overlaps with Lin et al. 2018 NHIS, and after discussing with study authors Lin et al. 2018 felt most appropriate^13^ |
| Yen et al. 2017^46^ | 46,028 | General population | Overlaps with Lin et al. 2018 NHIS, and after discussing with study authors Lin et al. 2018 felt most appropriate^13^ |
| Park et al. 2022^47^ | 2,396,434 | General population | Overlaps with Cho et al. 2022^12^ and Choi et al. 2021^17^, which were much larger and more appropriately investigated exposure of interest. Park et al. 2022^47^ investigated duration of underweight in previous years prior to the study. |
| Were et al. 2009^48^ | 1,015 | People with HIV | Overlaps with Moore et al. 2007^41^, which provides data in a more accessible format. |
| Long et al. 2020^49^ | 1,788 | People with rheumatological diseases | Wrong population |
| Jung et al. 2016^50^ | 1,776 | People post-gastrectomy | Wrong population |
| Beshir et al. 2019^51^ | 428 | Children | Wrong population |
| Okwara et al. 2017^52^ | 414 | Children | Wrong population |
| Hanrahan et al. 2010^53^ | 3,456 | People with HIV | Cases and person-years not available |
| Ganesan et al. 2023^54^ | 3,171 | People with HIV | Cases and person-years not available |
| Getu et al. 2022^55^ | 529 | People with HIV | Cases and person-years not available |
| Worodria et al 2011^56^ | 225 | People with HIV | Cases and person-years not available |
| Gatechompol et al. 2022^57^ | 2,849 | People with HIV | Cases and person-years not available |
| Pealing et al. 2015^58^ | 1,441,347 | General population | Cases and person-years not available |
| Liu et al. 2015^59^ | 67,686 | People with HIV | Cases and person-years not available |
| Chan-Yeung et al. 2007^60^ | 3,605 | General population | Could not access full text and cases and person-years not available |
| **Cohorts included in our meta-analyses not included in Franco et al. 2024 meta-analyses (additional participants excluding overlap and cohorts excluded for bias = 3,008,914)** | | | |
| Cho et al. 2022^12^ | 11,135,332 | General population | Excluded as they chose to include Park et al. 2022^47^ |
| Choi et al. 2021^17^ | 10,087,903 | General population | Excluded as they chose to include Park et al. 2022^47^ |
| Lin et al. 2018 NHIS^13^ | 48,713 | General population | Excluded as they chose to include Baker et al. 2012^45^ and Yen et al. 2017^46^ |
| Cheng et al. 2020^15^ | 34,076 | General population | Excluded from meta-analyses because of risk of bias |
| Soh et al. 2019^19^ | 50,398 | General population | Excluded from meta-analyses because of risk of bias |
| Larsson et al. 2025^23^ | 2,107 | General population | Not published at time |
| Nguenha et al. 2025^33^ | 3,593 | People with HIV | Not published at time |
| Jiang et al. 2024^18^ | 39,122 | General population | Not published at time |
| Sinha et al. 2024^25^ | 857 | General population | Not published at time |
| Saunders et al. 2017^21^ | 1,767 | General population | Excluded as BMI analysed as a continuous variable. Re-analysed for our study. |
| Saunders et al. 2020^22^ | 11,605 | General population | Excluded as BMI analysed as a continuous variable. Re-analysed for our study. |
| Mupfumi et al. 2018^34^ | 240 | People with HIV | Excluded as BMI analysed as a continuous variable. Re-analysed for our study. |
| Tverdal 1986^9^ | 1,717,655 | General population | Excluded as wrong "undernutrition" category |
| Kufa et al. 2016^40^ | 634 | People with HIV | Excluded as insufficient adjustment |
| Li et al. 2020^43^ | 234,418 | People with diabetes | Excluded as insufficient adjustment |
| Hemila et al. 1989^8^ | 26,975 | General population | Not noted why excluded |
| Palmer et al. 1957^6^ | 68,754 | General population | Not noted why excluded |
| Edwards et al. 1971^7^ | 823,199 | General population | Not noted why excluded |
| Aemro et al. 2020^38^ | 494 | People with HIV | Not noted why excluded |
| Maokola et al. 2021^30^ | 75,812 | People with HIV | Not noted why excluded |
| Salvadori et al. 2015^31^ | 1,682 | People with HIV | Not noted why excluded |

NHIS; National Health Interview Surveys

**Figure S1.** Dose-response meta-analyses fitting linear and piecewise linear models for the relationship between body mass index and tuberculosis risk in a) general population cohorts; b) people with HIV; and c) people with diabetes.


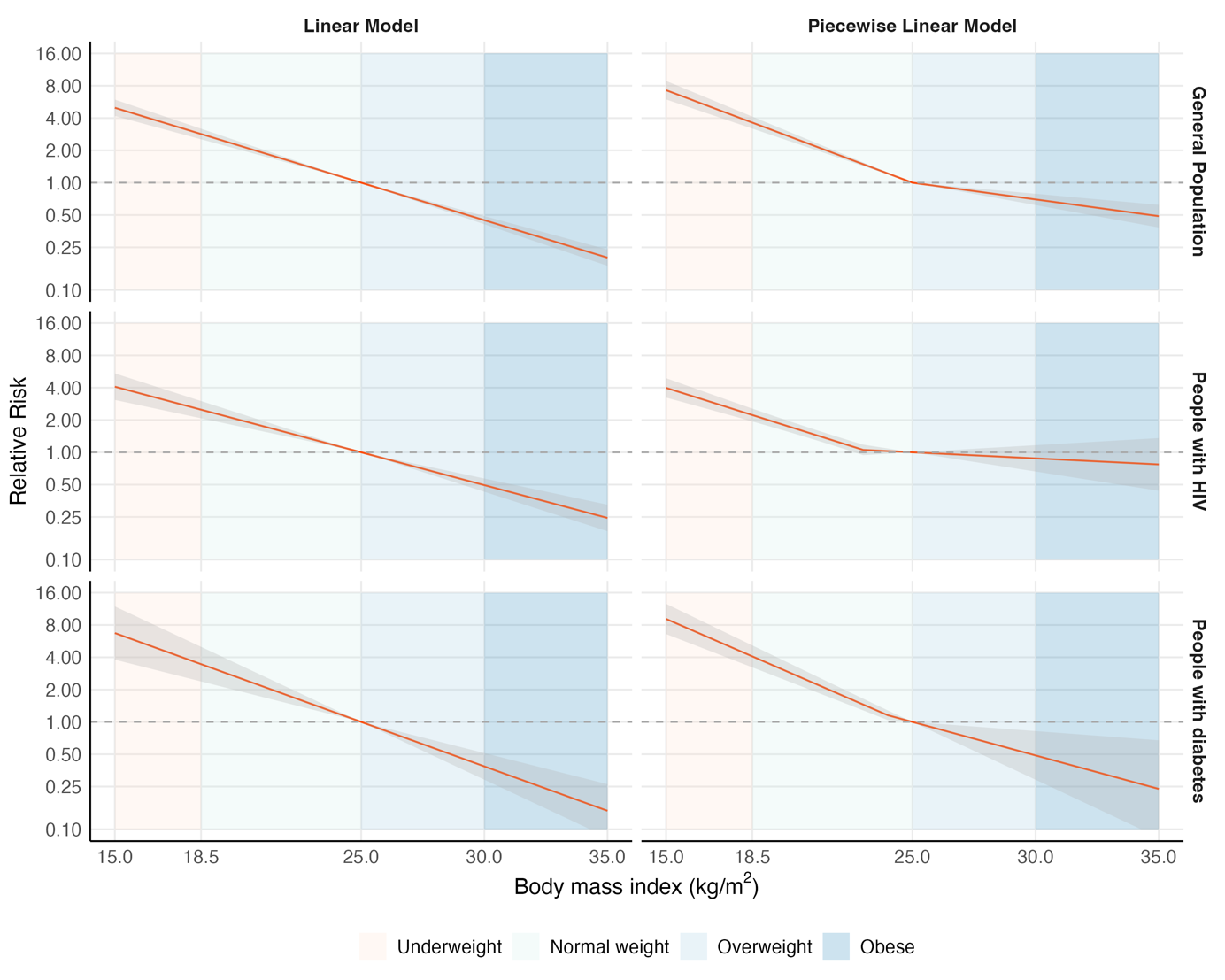


General population cohorts (22 cohorts, n=24,921,531); people with HIV (18 cohorts, n=162,609); people with diabetes (3 cohorts, n=1,118,424). See Table 1 for characteristics of included cohorts. The red line represents predictions of relative risk from the models with reference to a body mass index of 25.0kg/m^2^, and the shaded grey area represents 95% confidence intervals. In the linear models, the reduction in tuberculosis risk per one unit increase in body mass index was 14.8% (95%CI: 13.3-16.3) in the general population cohorts; 13.1% (95%CI: 10.6-15.6) in people with HIV, and 17.4% (95%CI: 12.5-21.9) in people with diabetes

**Figure S2.** Dose-response meta-analyses fitting linear, piecewise linear, and restricted cubic spline models for the relationship between body mass index and tuberculosis risk in general population cohorts in lower tuberculosis burden countries and high tuberculosis burden countries.


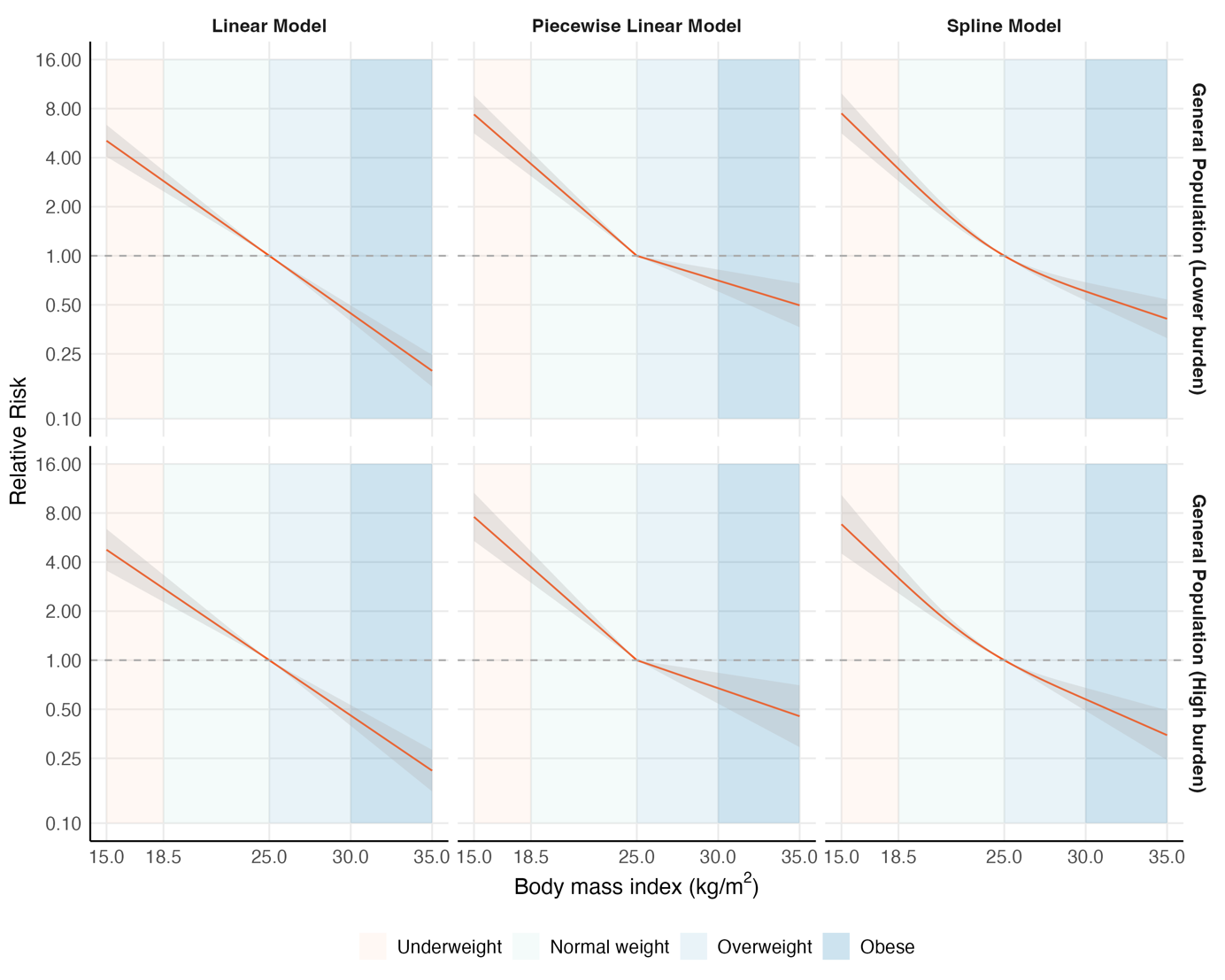


General population cohorts in lower burden countries (13 cohorts, n=24,797,722); General population cohorts in high burden countries (9 cohorts, n=123,809). See Table 1 for characteristics of included cohorts. The red line represents predictions of relative risk from the models with reference to a body mass index of 25.0kg/m^2^, and the shaded grey area represents 95% confidence intervals. For the piecewise linear models, the reduction in tuberculosis risk per one unit increase in body mass index was 18.1% (95%CI: 15.9-20.3) for <25.0kg/m^2^ and 6.8% (95%CI: 3.8-9.6) for ≥25.0kg/m^2^ in lower tuberculosis burden countries, and 18.3% (95%CI: 15.5-21.1) for <25.0kg/m^2^ and 7.6% (95%CI: 3.5-11.6) for ≥25.0kg/m^2^ in in high tuberculosis burden countries. For the linear models, the reduction in tuberculosis risk per one unit increase in body mass index was 15.0% (95%CI: 13.1-16.9) in lower tuberculosis burden countries and 14.5% (95%CI: 11.9-16.9) in high tuberculosis burden countries.

**REFERENCES FOR SUPPLEMENTARY MATERIAL**

1. Franco JV, Bongaerts B, Metzendorf MI, et al. Undernutrition as a risk factor for tuberculosis disease. *Cochrane Database Syst Rev* 2024; **6**(6): CD015890.

2. Lonnroth K, Williams BG, Cegielski P, Dye C. A consistent log-linear relationship between tuberculosis incidence and body mass index. *Int J Epidemiol* 2010; **39**(1): 149-55.

3. Collaboration NCDRF. Worldwide trends in body-mass index, underweight, overweight, and obesity from 1975 to 2016: a pooled analysis of 2416 population-based measurement studies in 128.9 million children, adolescents, and adults. *Lancet* 2017; **390**(10113): 2627-42.

4. World Health Organization. <https://www.who.int/data/gho/data/themes/topics/topic-details/GHO/body-mass-index> (accessed 30th March 2025).

5. Aibana O, Acharya X, Huang C-C, et al. Nutritional Status and Tuberculosis Risk in Adult and Pediatric Household Contacts. *PLoS One* 2016; **11**(11): e0166333.

6. Palmer CE, Jablon S, Edwards PQ. Tuberculosis morbidity of young men in relation to tuberculin sensitivity and body build. *Am Rev Tuberc* 1957; **76**(4): 517-39.

7. Edwards LB, Livesay VT, Acquaviva FA, Palmer CE. Height, weight, tuberculous infection, and tuberculous disease. *Arch Environ Health* 1971; **22**(1): 106-12.

8. Hemila H, Kaprio J, Pietinen P, Albanes D, Heinonen OP. Vitamin C and other compounds in vitamin C rich food in relation to risk of tuberculosis in male smokers. *Am J Epidemiol* 1999; **150**(6): 632-41.

9. Tverdal A. Body mass index and incidence of tuberculosis. *Eur J Respir Dis* 1986; **69**(5): 355-62.

10. Leung CC, Lam TH, Chan WM, et al. Lower risk of tuberculosis in obesity. *Arch Intern Med* 2007; **167**(12): 1297-304.

11. Cegielski JP, Arab L, Cornoni-Huntley J. Nutritional risk factors for tuberculosis among adults in the United States, 1971-1992. *Am J Epidemiol* 2012; **176**(5): 409-22.

12. Cho SH, Lee H, Kwon H, et al. Association of underweight status with the risk of tuberculosis: a nationwide population-based cohort study. *Sci Rep* 2022; **12**(1): 16207.

13. Lin H-H, Wu C-Y, Wang C-H, et al. Association of Obesity, Diabetes, and Risk of Tuberculosis: Two Population-Based Cohorts. *Clin Infect Dis* 2018; **66**(5): 699-705.

14. Chen J, Zha S, Hou J, et al. Dose-response relationship between body mass index and tuberculosis in China: a population-based cohort study. *BMJ Open* 2022; **12**(3): e050928.

15. Cheng J, Sun Y-N, Zhang C-Y, et al. Incidence and risk factors of tuberculosis among the elderly population in China: a prospective cohort study. *Infect Dis Poverty* 2020; **9**(1): 13.

16. Kim SJ, Ye S, Ha E, Chun EM. Association of body mass index with incident tuberculosis in Korea. *PLoS One* 2018; **13**(4): e0195104.

17. Choi H, Yoo JE, Han K, et al. Body Mass Index, Diabetes, and Risk of Tuberculosis: A Retrospective Cohort Study. *Front Nutr* 2021; **8**: 739766.

18. Jiang H, Chen X, Lv J, et al. Prospective cohort study on tuberculosis incidence and risk factors in the elderly population of eastern China. *Heliyon* 2024; **10**(3): e24507.

19. Soh AZ, Chee CBE, Wang YT, Yuan JM, Koh WP. Diabetes and body mass index in relation to risk of active tuberculosis: a prospective population-based cohort. *Int J Tuberc Lung Dis* 2019; **23**(12): 1277-82.

20. Yoo JE, Kim D, Choi H, et al. Anemia, sarcopenia, physical activity, and the risk of tuberculosis in the older population: a nationwide cohort study. *Ther Adv Chronic Dis* 2021; **12**: 20406223211015959.

21. Saunders MJ, Wingfield T, Tovar MA, et al. A score to predict and stratify risk of tuberculosis in adult contacts of tuberculosis index cases: a prospective derivation and external validation cohort study. *Lancet Infect Dis* 2017; **17**(11): 1190-9.

22. Saunders MJ, Wingfield T, Datta S, et al. A household-level score to predict the risk of tuberculosis among contacts of patients with tuberculosis: a derivation and external validation prospective cohort study. *Lancet Infect Dis* 2020; **20**(1): 110-22.

23. Larsson L, Calderwood CJ, Marambire E, et al. Body Mass Index Trajectories and Association with Tuberculosis Risk in a Cohort of Household Contacts in Southern Africa. Available at SSRN; 2024.

24. Paradkar M, Padmapriyadarsini C, Jain D, et al. Tuberculosis preventive treatment should be considered for all household contacts of pulmonary tuberculosis patients in India. *PLoS One* 2020; **15**(7): e0236743.

25. Sinha P, Ezhumalai K, Du X, et al. Undernourished Household Contacts Are at Increased Risk of Tuberculosis (TB) Disease, but not TB Infection-a Multicenter Prospective Cohort Analysis. *Clin Infect Dis* 2024; **79**(1): 233-6.

26. Batista JdAL, de Albuquerque MdFPM, Maruza M, et al. Incidence and risk factors for tuberculosis in people living with HIV: cohort from HIV referral health centers in Recife, Brazil. *PLoS One* 2013; **8**(5): e63916.

27. Tchakounte Youngui B, Coffie P, Messou E, et al. Incidence of Tuberculosis During the First Year of Antiretroviral Treatment in West African HIV-Infected Adults. *Open Forum Infect Dis* 2020; **7**(6): ofaa203.

28. Kyaw NTT, Kumar AMV, Harries AD, et al. Synergy between low BMI and hyperglycemia at baseline increases tuberculosis incidence among people living with HIV. *AIDS* 2022; **36**(1): 117-25.

29. Maro I, Lahey T, MacKenzie T, et al. Low BMI and falling BMI predict HIV-associated tuberculosis: a prospective study in Tanzania. *Int J Tuberc Lung Dis* 2010; **14**(11): 1447-53.

30. Maokola WM, Ngowi BJ, Mahande MJ, Todd J, Robert M, Msuya SE. Impact of Isoniazid Preventive Therapy on Tuberculosis incidence among people living with HIV: A secondary data analysis using Inverse Probability Weighting of individuals attending HIV care and treatment clinics in Tanzania. *PLoS One* 2021; **16**(7 July): e0254082.

31. Salvadori N, Chalermpantmetagul S, Figoni J, et al. Incidence of active tuberculosis in HIV-infected adults and mortality in Thailand. *Top Antivir Med* 2015; **23**(E-1): 377.

32. Chang CA, Meloni ST, Eisen G, et al. Tuberculosis Incidence and Risk Factors Among Human Immunodeficiency Virus (HIV)-Infected Adults Receiving Antiretroviral Therapy in a Large HIV Program in Nigeria. *Open Forum Infect Dis* 2015; **2**(4): ofv154.

33. Nguenha D, Ndebele F, Saavedra B, et al. BMI as a predictor of progression from TB infection to active TB in PLHIV. *Int J Tuberc Lung Dis* 2025; **29**(2): 54-9.

34. Mupfumi L, Moyo S, Molebatsi K, et al. Immunological non-response and low hemoglobin levels are predictors of incident tuberculosis among HIV-infected individuals on Truvada-based therapy in Botswana. *PLoS One* 2018; **13**(1): e0192030.

35. Alemu A, Yesuf A, Zerihun B, Getu M, Worku T, Bitew ZW. Incidence and determinants of tuberculosis among HIV-positive individuals in Addis Ababa, Ethiopia: A retrospective cohort study. *Int J Infect Dis* 2020; **95**: 59-66.

36. Tiruneh G, Getahun A, Adeba E. Assessing the Impact of Isoniazid Preventive Therapy (IPT) on Tuberculosis Incidence and Predictors of Tuberculosis among Adult Patients Enrolled on ART in Nekemte Town, Western Ethiopia: A Retrospective Cohort Study. *Interdiscip Perspect Infect Dis* 2019; **2019**: 1413427.

37. Ahmed A, Mekonnen D, Shiferaw AM, Belayneh F, Yenit MK. Incidence and determinants of tuberculosis infection among adult patients with HIV attending HIV care in north-east Ethiopia: a retrospective cohort study. *BMJ Open* 2018; **8**(2): e016961.

38. Aemro A, Jember A, Anlay DZ. Incidence and predictors of tuberculosis occurrence among adults on antiretroviral therapy at Debre Markos referral hospital, Northwest Ethiopia: retrospective follow-up study. *BMC Infect Dis* 2020; **20**(1): 245.

39. Nicholas S, Sabapathy K, Ferreyra C, Varaine F, Pujades-Rodriguez M. Incidence of tuberculosis in HIV-infected patients before and after starting combined antiretroviral therapy in 8 sub-Saharan African HIV programs. *J Acquir Immune Defic Syndr* 2011; **57**(4): 311-8.

40. Kufa T, Chihota V, Mngomezulu V, et al. The incidence of tuberculosis among hiv-positive individuals with high CD4 counts: implications for policy. *BMC Infect Dis* 2016; **16**: 266.

41. Moore D, Liechty C, Ekwaru P, et al. Prevalence, incidence and mortality associated with tuberculosis in HIV-infected patients initiating antiretroviral therapy in rural Uganda. *AIDS* 2007; **21**(6): 713-9.

42. Choun K, Thai S, Pe R, Lorent N, Lynen L, van Griensven J. Incidence and risk factors for tuberculosis in HIV-infected patients while on antiretroviral treatment in Cambodia. *Trans R Soc Trop Med Hyg* 2013; **107**(4): 235-42.

43. Li Y, Guo J, Xia T, et al. Incidence of pulmonary tuberculosis in Chinese adults with type 2 diabetes: a retrospective cohort study in Shanghai. *Sci Rep* 2020; **10**(1): 8578.

44. Gedfew M, Ayana M, Abate A, et al. Incidence and Predictors of Tuberculosis among Adult Diabetic Patients, Debre Markos Referral Hospital, Northwest Ethiopia, 2018: A Retrospective Cohort Study. *Diabetes Metab Syndr Obes* 2020; **13**: 869-78.

45. Baker MA, Lin H-H, Chang H-Y, Murray MB. The risk of tuberculosis disease among persons with diabetes mellitus: a prospective cohort study. *Clin Infect Dis* 2012; **54**(6): 818-25.

46. Yen YF, Hu HY, Lee YL, et al. Obesity/overweight reduces the risk of active tuberculosis: a nationwide population-based cohort study in Taiwan. *Int J Obes* 2017; **41**(6): 971-5.

47. Park J, Yoon JH, Ki HK, Eun Y, Han K, Kim H. Association of duration of undernutrition with occurrence of tuberculosis. *BMC Public Health* 2022; **22**(1): 2392.

48. Were W, Moore D, Ekwaru P, et al. A simple screening tool for active tuberculosis in HIV-infected adults receiving antiretroviral treatment in Uganda. *Int J Tuberc Lung Dis* 2009; **13**(1): 47-53.

49. Long W, Cai F, Wang X, Zheng N, Wu R. High risk of activation of latent tuberculosis infection in rheumatic disease patients. *Infect Dis* 2020; **52**(2): 80-6.

50. Jung WJ, Park YM, Song JH, et al. Risk factors for tuberculosis after gastrectomy in gastric cancer. *World J Gastroenterol* 2016; **22**(8): 2585-91.

51. Beshir MT, Beyene AH, Tlaye KG, Demelew TM. Incidence and predictors of tuberculosis among HIV-positive children at Adama Referral Hospital and Medical College, Oromia, Ethiopia: a retrospective follow-up study. *Epidemiol Health* 2019; **41**: e2019028.

52. Okwara FN, Oyore JP, Were FN, Gwer S. Correlates of isoniazid preventive therapy failure in child household contacts with infectious tuberculosis in high burden settings in Nairobi, Kenya - a cohort study. *BMC Infect Dis* 2017; **17**(1): 623.

53. Hanrahan CF, Golub JE, Mohapi L, et al. Body mass index and risk of tuberculosis and death. *AIDS* 2010; **24**(10): 1501-8.

54. Ganesan K, Mwesigwa R, Dear N, et al. Epidemiology of Tuberculosis Among People Living With HIV in the African Cohort Study From 2013 to 2021. *J Acquir Immune Defic Syndr* 2023; **92**(5): 359-69.

55. Getu A, Wolde HF, Animut Y, Kibret AA. Incidence and predictors of Tuberculosis among patients enrolled in Anti-Retroviral Therapy after universal test and treat program, Addis Ababa, Ethiopia. A retrospective follow -up study. *PloS One* 2022; **17**(8): e0272358.

56. Worodria W, Massinga-Loembe M, Mayanja-Kizza H, et al. Antiretroviral treatment-associated tuberculosis in a prospective cohort of HIV-infected patients starting ART. *Clin Dev Immunol* 2011; **2011**: 758350.

57. Gatechompol S, Sophonphan J, Ubolyam S, et al. Incidence and factors associated with active tuberculosis among people living with HIV after long-term antiretroviral therapy in Thailand: a competing risk model. *BMC Infect Dis* 2022; **22**(1): 346.

58. Pealing L, Wing K, Mathur R, Prieto-Merino D, Smeeth L, Moore DAJ. Risk of tuberculosis in patients with diabetes: population based cohort study using the UK Clinical Practice Research Datalink. *BMC Med* 2015; **13**: 135.

59. Liu E, Makubi A, Drain P, et al. Tuberculosis incidence rate and risk factors among HIV-infected adults with access to antiretroviral therapy. *AIDS* 2015; **29**(11): 1391-9.

60. Chan-Yeung M, Dai DL, Cheung AH, et al. Tuberculin skin test reaction and body mass index in old age home residents in Hong Kong. *J Am Geriatr Soc* 2007; **55**(10): 1592-7.

**PRISMA checklist.**

| **Section and Topic** | **Item #** | **Checklist item** | **Location where item is reported** |
| --- | --- | --- | --- |
| **TITLE** | | |  |
| Title | 1 | Identify the report as a systematic review. | Page 1 |
| **ABSTRACT** | | |  |
| Abstract | 2 | See the PRISMA 2020 for Abstracts checklist. | Page 2 |
| **INTRODUCTION** | | |  |
| Rationale | 3 | Describe the rationale for the review in the context of existing knowledge. | Page 4 |
| Objectives | 4 | Provide an explicit statement of the objective(s) or question(s) the review addresses. | Page 4 |
| **METHODS** | | |  |
| Eligibility criteria | 5 | Specify the inclusion and exclusion criteria for the review and how studies were grouped for the syntheses. | Pages 6-8 and supplementary material |
| Information sources | 6 | Specify all databases, registers, websites, organisations, reference lists and other sources searched or consulted to identify studies. Specify the date when each source was last searched or consulted. | Pages 6-8 and supplementary material |
| Search strategy | 7 | Present the full search strategies for all databases, registers and websites, including any filters and limits used. | Pages 6-8 and supplementary material |
| Selection process | 8 | Specify the methods used to decide whether a study met the inclusion criteria of the review, including how many reviewers screened each record and each report retrieved, whether they worked independently, and if applicable, details of automation tools used in the process. | Pages 6-8 and supplementary material |
| Data collection process | 9 | Specify the methods used to collect data from reports, including how many reviewers collected data from each report, whether they worked independently, any processes for obtaining or confirming data from study investigators, and if applicable, details of automation tools used in the process. | Pages 6-8 and supplementary material |
| Data items | 10a | List and define all outcomes for which data were sought. Specify whether all results that were compatible with each outcome domain in each study were sought (e.g. for all measures, time points, analyses), and if not, the methods used to decide which results to collect. | Pages 6-8 and supplementary material |
|  | 10b | List and define all other variables for which data were sought (e.g. participant and intervention characteristics, funding sources). Describe any assumptions made about any missing or unclear information. | Pages 6-8 and supplementary material |
| Study risk of bias assessment | 11 | Specify the methods used to assess risk of bias in the included studies, including details of the tool(s) used, how many reviewers assessed each study and whether they worked independently, and if applicable, details of automation tools used in the process. | Bias not formally assessed |
| Effect measures | 12 | Specify for each outcome the effect measure(s) (e.g. risk ratio, mean difference) used in the synthesis or presentation of results. | Pages 6-8 and supplementary material |
| Synthesis methods | 13a | Describe the processes used to decide which studies were eligible for each synthesis (e.g. tabulating the study intervention characteristics and comparing against the planned groups for each synthesis (item #5)). | Pages 6-8 and supplementary material |
|  | 13b | Describe any methods required to prepare the data for presentation or synthesis, such as handling of missing summary statistics, or data conversions. | Pages 6-8 and supplementary material |
|  | 13c | Describe any methods used to tabulate or visually display results of individual studies and syntheses. | Pages 6-8 and supplementary material |
|  | 13d | Describe any methods used to synthesize results and provide a rationale for the choice(s). If meta-analysis was performed, describe the model(s), method(s) to identify the presence and extent of statistical heterogeneity, and software package(s) used. | Pages 6-8 and supplementary material |
|  | 13e | Describe any methods used to explore possible causes of heterogeneity among study results (e.g. subgroup analysis, meta-regression). | Pages 6-8 and supplementary material |
|  | 13f | Describe any sensitivity analyses conducted to assess robustness of the synthesized results. | Pages 6-8 and supplementary material |
| Reporting bias assessment | 14 | Describe any methods used to assess risk of bias due to missing results in a synthesis (arising from reporting biases). | Bias not formally assessed |
| Certainty assessment | 15 | Describe any methods used to assess certainty (or confidence) in the body of evidence for an outcome. | Pages 6-8 and supplementary material |
| **RESULTS** | | |  |
| Study selection | 16a | Describe the results of the search and selection process, from the number of records identified in the search to the number of studies included in the review, ideally using a flow diagram. | Pages 9-10; tables and figures; and supplementary material |
|  | 16b | Cite studies that might appear to meet the inclusion criteria, but which were excluded, and explain why they were excluded. | N/A |
| Study characteristics | 17 | Cite each included study and present its characteristics. | Pages 9-10; tables and figures; and supplementary material |
| Risk of bias in studies | 18 | Present assessments of risk of bias for each included study. | N/A |
| Results of individual studies | 19 | For all outcomes, present, for each study: (a) summary statistics for each group (where appropriate) and (b) an effect estimate and its precision (e.g. confidence/credible interval), ideally using structured tables or plots. | Pages 9-10; tables and figures; and supplementary material |
| Results of syntheses | 20a | For each synthesis, briefly summarise the characteristics and risk of bias among contributing studies. | Bias not formally assessed |
|  | 20b | Present results of all statistical syntheses conducted. If meta-analysis was done, present for each the summary estimate and its precision (e.g. confidence/credible interval) and measures of statistical heterogeneity. If comparing groups, describe the direction of the effect. | Pages 9-10; tables and figures; and supplementary material |
|  | 20c | Present results of all investigations of possible causes of heterogeneity among study results. | Pages 9-10; tables and figures; and supplementary material |
|  | 20d | Present results of all sensitivity analyses conducted to assess the robustness of the synthesized results. | Pages 9-10; tables and figures; and supplementary material |
| Reporting biases | 21 | Present assessments of risk of bias due to missing results (arising from reporting biases) for each synthesis assessed. | N/A |
| Certainty of evidence | 22 | Present assessments of certainty (or confidence) in the body of evidence for each outcome assessed. | N/A |
| **DISCUSSION** | | |  |
| Discussion | 23a | Provide a general interpretation of the results in the context of other evidence. | Pages 11-14 |
|  | 23b | Discuss any limitations of the evidence included in the review. | Pages 11-14 |
|  | 23c | Discuss any limitations of the review processes used. | Pages 11-14 |
|  | 23d | Discuss implications of the results for practice, policy, and future research. | Pages 11-14 |
| **OTHER INFORMATION** | | |  |
| Registration and protocol | 24a | Provide registration information for the review, including register name and registration number, or state that the review was not registered. | We did not register this review or prepare a protocol. |
|  | 24b | Indicate where the review protocol can be accessed, or state that a protocol was not prepared. |  |
|  | 24c | Describe and explain any amendments to information provided at registration or in the protocol. |  |
| Support | 25 | Describe sources of financial or non-financial support for the review, and the role of the funders or sponsors in the review. | Page 15 |
| Competing interests | 26 | Declare any competing interests of review authors. | Page 15 |
| Availability of data, code and other materials | 27 | Report which of the following are publicly available and where they can be found: template data collection forms; data extracted from included studies; data used for all analyses; analytic code; any other materials used in the review. | Page 15 |
